# Transcriptomic Analysis of *Oenococcus oeni* SD-2a Response to Acid Shock by RNA-Seq

**DOI:** 10.3389/fmicb.2017.01586

**Published:** 2017-08-22

**Authors:** Longxiang Liu, Hongyu Zhao, Shuai Peng, Tao Wang, Jing Su, Yanying Liang, Hua Li, Hua Wang

**Affiliations:** ^1^College of Enology, Northwest A&F University Yangling, China; ^2^College of Bioengineering, Sichuan University of Science and Engineering Zigong, China; ^3^College of Food Science and Engineering, Shanxi Agricultural University Taigu, China; ^4^Shaanxi Engineering Research Center for Viti-Viniculture Yangling, China; ^5^Heyang Experimental and Demonstrational Stations for Grape, Northwest A&F University Weinan, China

**Keywords:** *Oenococcus oeni*, transcriptomic, RNA-seq, acid shock, malolactic fermentation

## Abstract

*Oenococcus oeni* can be applied to conduct malolactic fermentation (MLF), but also is the main species growing naturally in wine. Due to the high stress tolerance, it is an interesting model for investigating acid response mechanisms. In this study, the changes in the transcriptome of *O.oeni* SD-2a during the adaptation period have been studied. RNA-seq was introduced for the transcriptomic analysis of *O. oeni* samples treated with pH 4.8 and pH 3.0 at 0 and 1 h, respectively. Gene ontology (GO) and Kyoto encyclopedia of genes and genome (KEGG) were performed to compare the transcriptome data between different treatments. From GO analysis, the majority of differentially expressed genes (DEGs) (pH 3.0_1 h-VS-pH 4.8_1 h, pH 3.0_1 h-VS-pH 4.8_0 h, and pH 4.8_1 h-VS-pH 4.8_0 h) were found to be involved in the metabolic process, catalytic activity, cellular process, and binding. KEGG analysis reveals that the most functional gene categories affected by acid are membrane transport, amino acid metabolism and carbohydrate metabolism. Some genes, like the heat shock protein Hsp20, malate transporter and malate permease, were also over-expressed in response to acid stress. In addition, a considerable proportion of gene indicate a significantly different expression in this study, are novel, which needs to be investigated further. These results provide a new viewpoint and crucial resource on the acid stress response in *O. oeni*.

## Introduction

Malolactic fermentation (MLF) is a biological process involved in winemaking, in which tart-tasting dicarboxylic malic acid, naturally present in grape must, is converted to softer-tasting monocarboxylic lactic acid and carbon dioxide by decarboxylation (Spano and Massa, 2006). Through MLF, *Oenococcus oeni can* bring stabilization, sensory impacts, and deacidification to most red wines, so MLF and *O. oeni* are crucial in the process of winemaking (Wang et al., 2015). *O. oeni* is the main lactic acid bacteria existing in MLF. MLF and the growth of *O. oeni* are clearly inhibited by several of the physiochemical properties of wine (Betteridge et al., 2015). The four main stress factors in wine affecting MLF are ethanol (10–16% v/v), low pH (3.0–3.5), SO_2_ (over 10 mg/L), and low temperature (can be below 12°C) (Spano and Massa, 2006; Betteridge et al., 2015; Olguin et al., 2015; Darsonval et al., 2016). Many efforts have been put to investigate the mechanism of stress response of *O.oeni* (Spano and Massa, 2006; Olguin et al., 2015).

Low pH appears as a crucial parameter that limits bacterial growth in wine (Fortier et al., 2003). Currently, several studies have been launched to understand how *O. oeni* response under acid stress conditions, such as membrane composition and fluidity, pH homeostasis, oxidative stress response, DNA, and protein damage repair (Darsonval et al., 2016). But the mechanism of stress adaption in *O. oeni* still needs a further research.

The transcriptome of *O. oeni* has been studied and quantified via traditional approaches, like hybridization, fingerprinting, and tilling microarrays (Marques et al., 2012; Olguin et al., 2015; Margalef-Català et al., 2016). According to the transcriptomics and proteome results, the mechanism of stress response in *O. oeni* is believed very complicated, which involves series of proteins (GroEL, GroES, etc.), genes (*dnaJ, dnaK*, and *hsp18*, etc.) and metabolic pathways (amino acid transport and metabolism, malate, and citrate metabolism, etc.) (Margalef-Català et al., 2016). Nevertheless, there are still some disadvantages of these techniques, for example, non-specific cross-hybridization usually cause a high background level which limits the detection range, the transcripts can be detected only with high copy number, and the total coverage of the transcripts are almost unknown (Liu et al., 2015). Additionally, it is difficult and arduous to normalize methods and compare the expression data from different experiment.

RNA-seq is a revolutionary method with many advantages, like rapidness, high precision, reproducibility, and low cost (Liu et al., 2015). This technique is mainly applied to study the transcriptome differences from different treatments. The complexity, plasticity, and regulation of bacterial transcriptomes have been gradually appeared with the application of RNA-seq technology (Sorek and Cossart, 2010).

To provide genetic information on the acid response mechanisms of *O. oeni*, the transcriptome dataset was generated by using Illumina HiSeq™ 2500 platform. The transcriptomes of cells with and without acid stress were compared to determine the changes in the gene transcription level, as well as the functions and KEGG pathways of differentially expressed genes (DEGs) were analyzed. The RNA-seq data and the expression patterns are valuable genetic resources, that can advance knowledge on acid stress response of *O.oeni* or other bacteria's. Understanding the stress response mechanisms may help us to improve MLF starter robustness without using genetic engineering. Several works were done on stress mechanisms in *O.oeni* in wine-like medium /wine /microvinification etc. (Olguin et al., 2015; Margalef-Català et al., 2016). The originality, in this case, is the use of RNA-seq to investigate low pH response in *O.oeni*.

## Materials and methods

### Bacterial strain

The MLF starter used in this study is *O. oeni* SD-2a, which shows strong abilities to survive in stress conditions, and more active than commercial type strain (Viniflora® Oenos) in MLF ability. It was isolated from Chinese wines regions (Shandong province) and stored in College of Enology, Northwest A&F University (Liu, 2002; Wang et al., 2003; Zhang, 2008; Li et al., 2016). Many studies have been done on the commercial application of *O. oeni* SD-2a. The strain *O. oeni* SD-2a has obtained patent protection (02123444.2).

### Growth conditions

*O. oeni* SD-2a was cultured at 28°C in a flask containing FMATB broth medium at pH 4.8 (glucose 5 g/L, D, L-malate 5 g/L, yeast extract 5 g/L, peptone 10 g/L, MgSO_4_•7H_2_O 0.2 g/L, MnSO_4_•4H_2_O 0.05 g/L, Cysteine/HCl 0.5 g/L, and tomato juice 250 mL) (Li et al., 2009). When cultures reached the mid-exponential phase (OD600 nm ≈ 1) they were mixed and divided into six equal parts. Then cells were harvested by centrifugation (12, 000 rpm for 1 min at 25°C). Immediately, they were washed by FMATB broth medium at pH 3.0 and pH 4.8 (control) into a same sterile flask, respectively. The possible effect of centrifugation and time were evaluated using the control assay with pH 4.8. All assays were performed in triplicate using independent cultures and incubated at 28°C. Samples were taken at time zero just before acid shock, and then at one hour with or without acid shock (Margalef-Català et al., 2016).

### RNA extraction

Cells were harvested and kept by following the protocol of (Margalef-Català et al., 2016). Total RNA was extracted by using the RNAprep pure Cell/Bacteria Kit (Tiangen, Beijing, China) following the manufacturer's instructions. To determine the concentration of RNA, the absorbance at 260 nm was measured using a BioDrop μLITE Spectrophotometer (Tamar Laboratory Supllies LTD., Cambridge, England) (Margalef-Català et al., 2016). The RNA integrity number (RIN) and 28S:18S ratio were also measured, total RNA samples with RIN > 7.0 and a 28S:18S ratio > 1.8 were used in subsequent experiments (Miller et al., 2009).

### cDNA library construction and sequencing

Sequence libraries were generated and sequenced by CapitalBio Technology (Beijing, China). The triplicate samples of all assays were constructed an independent library, and do the following sequencing and analysis. The NEB Next Ultra RNA Library Prep Kit for Illumina (NEB) was used to construct the libraries for sequencing. NEB Next Poly(A) mRNA Magnetic Isolation Module (NEB) kit was used to enrich the poly(A) tailed mRNA molecules from 1 μg total RNA. The mRNA was fragmented into ~200 base pair pieces. The first-strand cDNA was synthesized from the mRNA fragments reverse transcriptase and random hexamer primers, and then the second-strand cDNA was synthesized using DNA polymerase I and RNaseH. The end of the cDNA fragment was subjected to an end repair process that included the addition of a single “A” base, followed by ligation of the adapters. Products were purified and enriched by polymerase chain reaction (PCR) to amplify the library DNA. The final libraries were quantified using KAPA Library Quantification kit (KAPA Biosystems, South Africa) and an Agilent 2100 Bioanalyzer. After quantitative reverse transcription-polymerase chain reaction (RT-qPCR) validation, libraries were subjected to paired-end sequencing with pair end 150-base pair reading length on an Illumina HiSeq sequencer (Illumina) (Kwon et al., 2016).

### RNA-seq: data analysis

The genome of *O. oeni* SD-2a was used as reference (unpublished). The sequencing quality were assessed with FastQC (Version 0.11.5) and then low quality data were filtered using NGSQC (v0.4).The clean reads were then aligned to the reference genome using HISAT2 (Johns Hopkins University, USA) with default parameters (Liu et al., 2015).

The processed reads from each sample were aligned using HISAT (Johns Hopkins University, USA) against the corresponding *O. oeni* SD-2a reference genome. The gene expression analyses were performed with Cuffquant and Cuffnorm (Cufflinks 2.2.1).

Cuffdiff was used to analyze the DEGs between samples. The standardization method of Cuffdiff is geometric, with the per-condition and pooled as the discrete model (Trapnell et al., 2013). Thousands of independent statistical hypothesis testing were conducted on DEGs, separately. Then a *p*-value was obtained, which was corrected by FDR method. And Corrected *P*-value (*q*-value) was calculated by correcting using BH method. *p*-value or *q*-value were used to conduct significance analysis. Parameters for classifying significantly DEGs are ≥2-fold differences (|log_2_FC|≥1, FC: the fold change of expressions) in the transcript abundance and *q* < 0.05 (Parreira et al., 2016).

By searching the ENSEMBL, NCBI, Uniprot, GO, and KEGG databases, the BLAST (Basic Local Alignment Search Tool) alignment was performed to determine the functional annotation of DEGs. The best matches were selected to annotate the DEGs. Finally, DEGs were subjected to GO functional analysis and KEGG, utilizing default parameters, to annotate the DEGs' major GO, and KEGG categories (Liu et al., 2014; Parreira et al., 2016).

### Validation of RNA-seq data by RT-qPCR

To validate the RNA-seq data, RT-qPCR was introduced. Several genes were selected for the validation. Some genes were selected due to their involvement in stress response according to previous studies (Beltramo et al., 2006; Olguin et al., 2009, 2015), and others were randomly selected (Table 1). The RNA samples used are same as used in RNA-seq analysis. The primers were selected and analyzed by the Primer Premier Software (version 5.0). In this work, five genes (*ldhD, dpoIII, dnaG, gyrA*, and *gyrB*) were evaluated as internal controls for RT-qPCR, using the primers described in Table 1 (Desroche et al., 2005; Costantini et al., 2011; Margalef-Català et al., 2016). The five internal controls were calculated on their geometric mean for the normalization of RT-qPCR data (Sumby et al., 2012). The Real Time PCR System iQ5 (Bio-Rad) was used for the amplification of RT-qPCR. The threshold value used in this study was automatically determined by the instrument. Results were analyzed using the 2^−ΔΔCT^ method, and the amount of target RNA was adjusted to the geometric mean of the five internal controls as previously described (Livak and Schmittgen, 2001).

**Table 1:** Gene descriptions and the corresponding primer sequences used for validation of RNA-seq results by RT-qPCR.

**Gene symbol**	**Tracking_id**	**Sequence(5′–3′)**	**References**
Heat-shock protein Hsp20 *(hsp18)*	orf00243	F-CGGTATCAGGAGTTTTGAGTTC	Beltramo et al., 2006
		R-CGTAGTAACTGCGGGAGTAATTC	
Malate transporter	orf01583	F-TTATCGGCATCTCAGTTCATACAGC	This work
		R-CAGACAAAACCCCAAGACTATCACG	
Membrane protein	orf00399	F-TGGTCTTGGAACGGCATTAGGCGA	This work
		R-ATCAGCAAATGAAGCACCGAGGGG	
*butA*(acetoin reductase)	orf00591	F-GGACTGATTGGTAGACATTTAGAA	This work
		R-GCGTTTTGAGACATCGGCTTTTTT	
F_0_F_1_ ATP synthase subunit gamma(*ATPF1G*)	orf00568	F-ATTCGTCGTCGGATTGATTC	This work
		R-CGAGATATCCGGACGTATGC	
Molecular chaperone DnaK(*dnaK*)	orf01216	F-CCGGTTTGAGCTTCTCTGAC	This work
		R-CGGGTTAATCGAATGGTTTG	
ATP dependent Clp protease proteolytic subunit (*clpP*)	orf00480	F-CGGTACCAAAGGCAAGCGTTTTAT	Beltramo et al., 2006
		R-CTCTTCCGAGTCTTCAAAAGTTGAT	
Citrate lyase(*citE*)	orf00341	F-CCGCACGATGATGTTTGTTCC	Olguin et al., 2009
		R-GCTCAAAGAAACGGCATCTTCC	
ATP-dependent protease(*clpX*)	orf01869	F-TTTGTGGTAAACGCCAGGAT	Beltramo et al., 2006
		R-TGCTCATGCTCCAGTTCTTG	
Nucleotide exchange factor GrpE(*grpE*)	orf01217	F-CGCAGGCAGAAAAGAACAATC	Beltramo et al., 2006
		R-GCTGAAGACGAAGCAGTTGC	
D-methionine transport system substrate-binding protein (*metQ*)	orf00953	F-CAGTCGGTTCTCAAGGTTCC	This work
		R-GCCCTGTGCTGTAGCCTTAT	
D-lactate dehydrogenase(*ldhD*)	orf00332	F-GCCGCAGTAAAGAACTTGATG	Margalef-Català et al., 2016
		R-TGCCGACAACACCAACTGTTT	
DNA polymerase III subunit alpha(*dpoIII*)	orf00690	F-GCAGTGAAGGGACGCTTAAACG	Costantini et al., 2011
		R-ACCCAATCGCCTCGACATCATC	
DNA primase(*dnaG*)	orf00886	F-TGTGGACGGAGTGGCAATGT	Desroche et al., 2005
		R-CGGTATTTTCTGTATATTTACTATCG	
DNA gyrase subunit A(*gyrA*)	orf02027	F-CGCCCGACAAACCGCATAAA	Desroche et al., 2005
		R-CAAGGACTCATAGATTGCCGAA	
DNA gyrase subunit B(*gyrB*)	orf02026	F-GAGGATGTCCGAGAAGGAATTA	Desroche et al., 2005
		R-GCCTGCTGGGCATCTGTATTA	

## Results and discussion

To better understand the stress response and regulation mechanism of *O. oeni*, functional analysis based on comparative transcriptomics was used in this study. The genes most affected by acid shock were mainly studied in this paper. mRNA from the control (pH 4.8) at t = 0 h and t = 1 h, and from acid treated samples at t = 1 h were used to conduct transcriptional analysis. The RNA-seq data using in this article have been submitted to Sequence Read Archive (SRA) database with an accession number of SRP105332.

**Figure 1:**
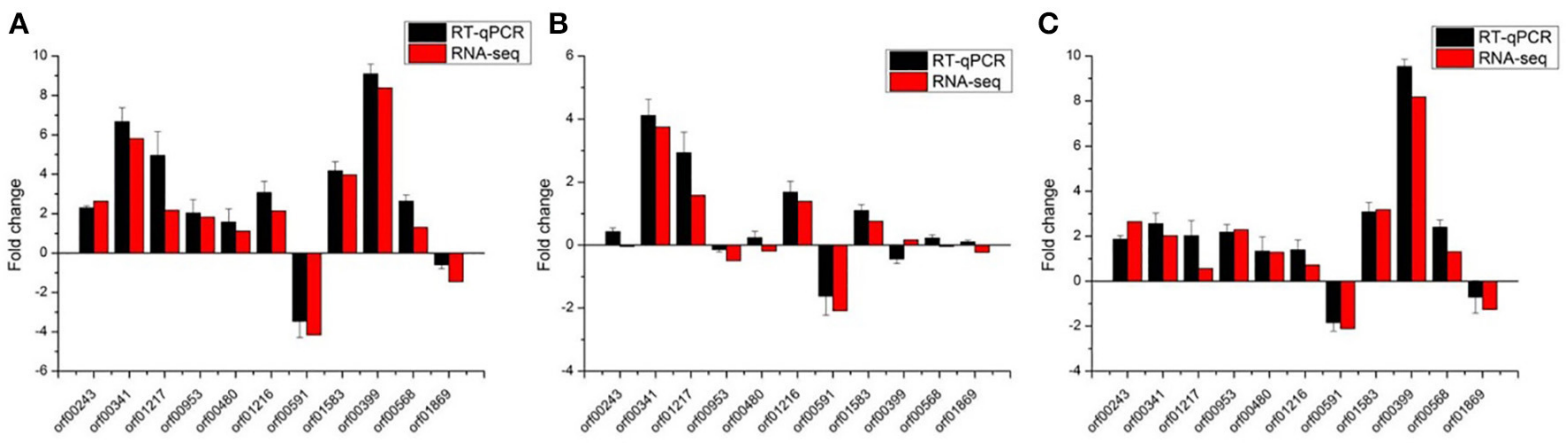
Validation of RNA-seq data using RT-qPCR with the internal control genes normalized by the geometric mean. Eleven representative genes were chosen to validate the RNA-Seq data by RT-qPCR. The black bars represent mean values of −ΔΔCT obtained from three biological replicates of RT-qPCR with error bars stand for standard deviations. The RT-qPCR data were normalized by the geometric mean of gene *dnaG, dpoIII, gyrA, gyrB*, and *ldhD*. And the red bars represent RNA-seq data. **(A–C)** Represent group VS1: pH 3.0_1 h-VS-pH 4.8_0 h, VS2: pH 4.8_1 h-VS-pH 4.8_0 h, and VS3: pH 3.0_1 h-VS-pH 4.8_1 h, respectively.

The results obtained from the RNA-seq were validated by RT-qPCR with the same RNA samples, and 11 genes were selected in this section (Table 1). For all the 11 genes tested, the RT-qPCR data have a general accordance with RNA-seq data (Figure 1 and Supplementary Figure 1). Of the 11 genes in different groups, most were clearly correlated using both techniques. Indicating no significant changes through this technique, although some genes display low correlated in the group VS2 (pH 4.8_1 h-VS-pH 4.8_0 h). Overall, the correlation between RT-qPCR and RNA-seq is good, suggesting that the RNA-seq data are valid.

### Global analysis of functions affected during acclimation after acid shock

In order to identify the biological processes influenced by acid shock, transcriptomic data were grouped by functional categories. pH 4.8_0 h and pH 4.8_1 h, as the reference conditions, were used to normalize data. Under the control conditions, the expression level of some genes was decreased, probably due to the influence of centrifugation (data not shown). However, acid shock is the biggest influencing factor in gene expression. **Table 4** shows some DEGs from each functional category after acid shock at pH 3.0 (t = 1 h). Genes within a wide range of functional classes were influenced by acid shock.

**Figure 2:**
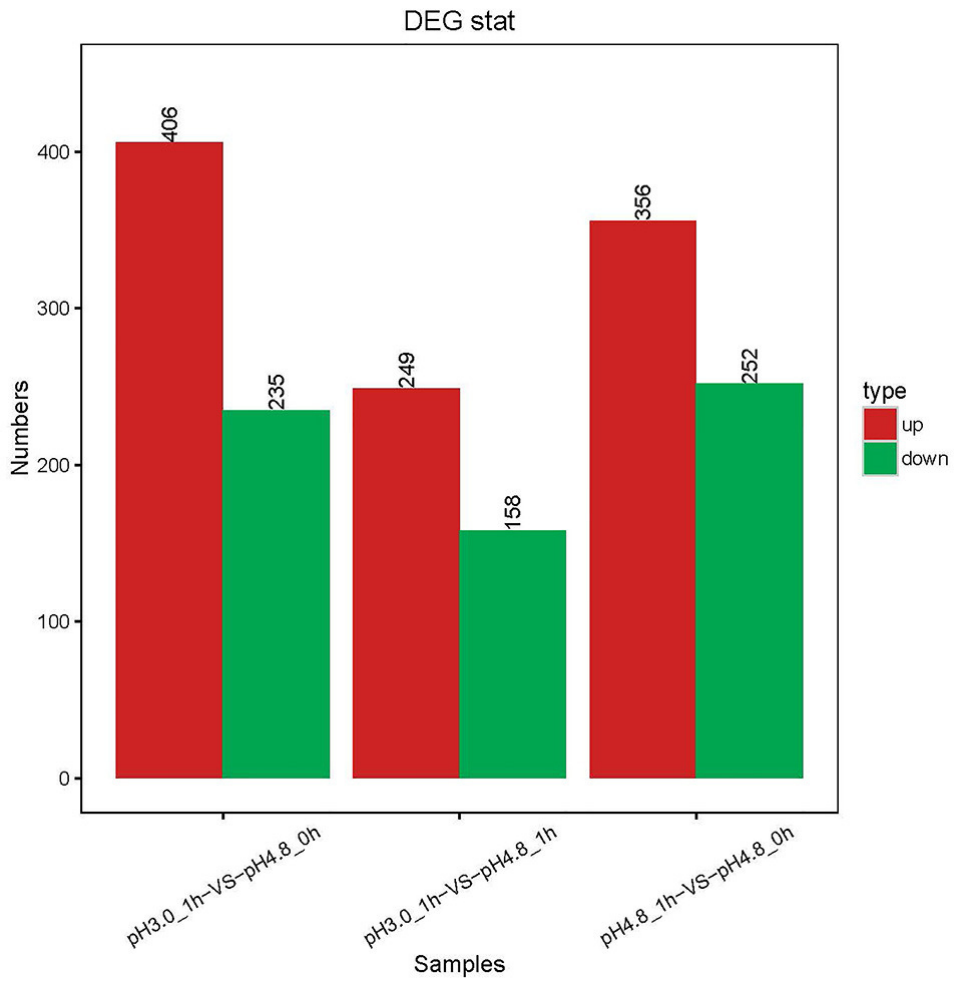
Changes in gene expression profile in the three comparisons. The red and green bars represent up- and down-regulated genes, respectively, and the numeric labels represent the number of genes in the group.

A total of 955 DEGs were detected by the RNA-seq. It is significantly higher than those identified by Margalef-Català et al. (2016). But in the three separate comparison groups, the numbers of DEGs were almost the same or less than that in Margalef-Català et al. (2016). Of these, as in Figure 2, compared to pH 4.8_0 h, 235 genes decreased their expression 1 h after acid shock and 406 genes increased their expressions. Compare to pH 4.8_1 h, 158 genes decreased in their expression after 1 h acid shock and 249 genes increased in their expression. Compared to the research of Margalef, apart the techniques, the media (WLM in the case of Margalef), strain (PSU-1 in the case of Margalef) were different as well. These differences could be corrected by the setting of control groups. The samples of pH 4.8_0 h and pH 4.8_1 h were set as control groups in this study, while the samples at 0 h were control groups in the case of Margalef. The DEGs number of comparisons VS1 (pH 3.0_1 h-VS-pH 4.8_0 h) was almost the same as Margalef, which was much higher than VS3 (pH 3.0_1 h-VS-pH 4.8_1 h). With the group of pH 4.8_0 h as control, the result may overlooked the genes differentially expressed due to the time changing, which is normal in the process of bacterial growth. Therefore, the group of pH 4.8_1 h was set as the primary control in this study.

**Figure 3:**
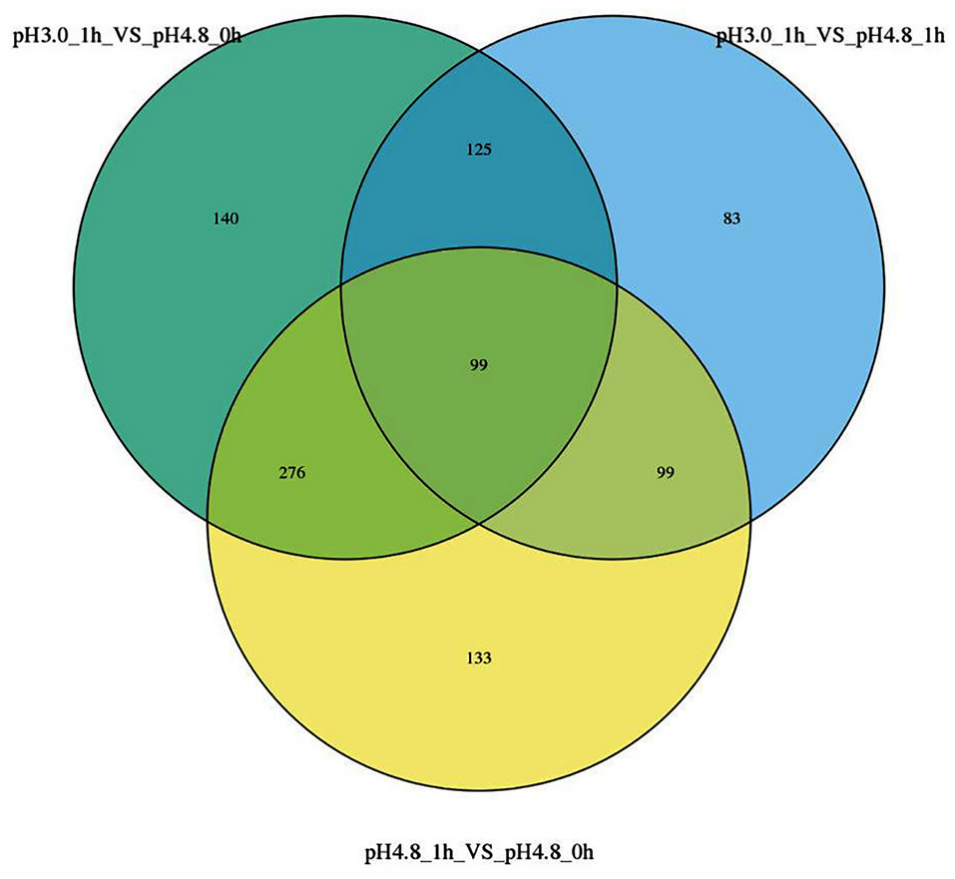
Venn diagrams show the overlaps among the three comparisons.

All the specific DEGs numbers changed by different conditions were shown in a Venn diagram (Figure 3). Ninety-nine genes were identified to be expressed in significant difference within all comparisons. The analysis of comparisons VS1 and VS2 had the same transcription patterns with 375 genes, while only 99 genes showed some differences in comparison VS3, which suggests that with different control samples, the transcription patterns are also exist differences.

**Figure 4:**
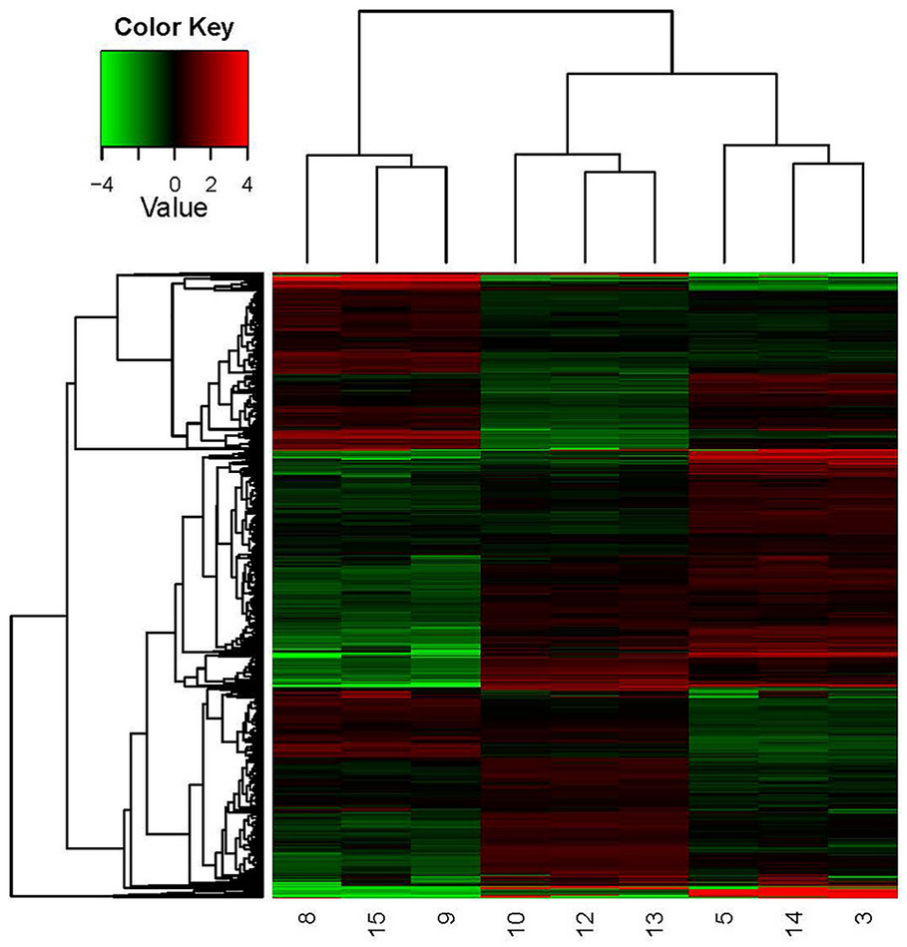
Hierarchical clustering in heat map format of all DEGs in *O. oeni* SD-2a grown under different conditions. Each horizontal row represents a differentially expressed gene, whereas each column represents a different growth condition. Green represents downregulated expression and red represents upregulated expression. Log2 values were used to cluster all the DEGs in Java TreeView by hierarchical clustering using Euclidean distance and pairwise average linkage methods. The number 9, 8, and 15 represent the three parallel samples of pH 4.8_0 h; The number 13, 12, and 10 represent the three parallel samples of pH 4.8_1 h; The number 3, 5, and 14 represent the three parallel samples of pH 3.0_1 h.

A hierarchical heat map (Figure 4) was adopted to show the global DEGs patterns occurring in the experimental conditions. The expression profiles under different growth conditions were shown in this map, obviously. In this study, the key factor influencing the cluster patterns of DEGs was growth condition. Thus, the DEGs patterns were similar within parallel samples, showing good correlation between parallel samples.

All the DEGs were also shown in the format of scatter diagram and volcano plot (Additional Supplementary Figures 2, 3).

### Functional analysis and classification of DEGs

To better understand the transcriptome of *O. oeni* SD-2a, the function of predicted genes was classified by GO and KEGG.

GO enrichment was used to identify the putative function of all the DEGs in every group, which can provides DEGs a statistical support in GO terms. In general, the enrichment analyses of DEGs showed that VS1, VS2, and VS3 were mainly belong to one category: biological processes (Table 2, Additional Supplementary Figures 4–6). Among them, the majority of DEGs of all groups (VS1, VS2, and VS3) were found to be involved in the metabolic process (GO:0008152), catalytic activity(GO:0003824), cellular process(GO:0009987), and binding(GO:0005488).

**Table 2:** Total number of differentially expressed genes enrichment by GO database.

**Class**	**GO term**	**GO name**	**VS1**	**VS2**	**VS3**
Molecular function	GO:0000988	Protein binding transcription factor activity	1	0	0
	GO:0001071	Nucleic acid binding transcription factor activity	14	13	6
	GO:0003824	Catalytic activity	256	255	180
	GO:0005198	Structural molecule activity	35	41	9
	GO:0005215	Transporter activity	51	42	34
	GO:0005488	Binding	195	196	120
	GO:0016209	Antioxidant activity	1	1	1
	GO:0030234	Enzyme regulator activity	1	1	0
	GO:0060089	Molecular transducer activity	1	1	1
	GO:0098772	Molecular function regulator	1	1	0
Cellular component	GO:0005576	Extracellular region	1	0	0
	GO:0005623	Cell	129	124	78
	GO:0016020	Membrane	92	86	60
	GO:0031012	Extracellular matrix	1	0	1
	GO:0032991	Macromolecular complex	56	57	23
	GO:0043226	Organelle	39	45	11
	GO:0044422	Organelle part	9	11	2
	GO:0044425	Membrane part	80	70	45
	GO:0044464	Cell part	129	124	78
Biological process	GO:0008152	Metabolic process	329	337	212
	GO:0009987	Cellular process	243	245	147
	GO:0022610	Biological adhesion	0	1	1
	GO:0023052	Signaling	5	2	4
	GO:0032502	Developmental process	3	2	1
	GO:0044699	Single-organism process	189	207	136
	GO:0048518	Positive regulation of biological process	0	0	1
	GO:0048519	Negative regulation of biological process	2	3	1
	GO:0050789	Regulation of biological process	39	36	24
	GO:0050896	Response to stimulus	19	21	13
	GO:0051179	Localization	85	75	53
	GO:0051704	Multi-organism process	1	2	0
	GO:0065007	Biological regulation	40	38	25
	GO:0071840	Cellular component organization or biogenesis	15	19	11

*VS1, is the comparison of pH 3.0_1 h-VS-pH 4.8_0 h; VS2, is the comparison of pH 4.8_1 h-VS-pH 4.8_0 h; VS3, is the comparison of pH 3.0_1 h-VS-pH 4.8_1 h*.

Among the KEGG enrichment of DEGs, some groups seemed to be less affected by acid (like transport and catabolism, metabolism of terpenoids, and polyketides, biosynthesis of other secondary metabolites, transcription and signal transduction mechanisms), while others were more sensitive (like amino acid metabolism, carbohydrate metabolism, membrane transport, and energy metabolism) (Table 3, Additional Supplementary Figures 7–9).

**Table 3:** Total number of differentially expressed genes in the KEGG pathway.

**KO term**	**VS1**	**VS2**	**VS3**
Aging	2	1	1
Amino acid metabolism	35	36	38
Biosynthesis of other secondary metabolites	6	4	6
Carbohydrate metabolism	79	65	44
Cell growth and death	2	2	3
Endocrine system	6	7	4
Energy metabolism	21	17	18
Folding, sorting and degradation	6	5	6
Global and overview maps	37	39	41
Glycan biosynthesis and metabolism	10	9	5
Lipid metabolism	13	20	11
Membrane transport	52	46	36
Metabolism of cofactors and vitamins	15	18	21
Metabolism of other amino acids	13	12	9
Metabolism of terpenoids and polyketides	10	10	5
Nucleotide metabolism	29	33	15
Replication and repair	8	8	4
Signal transduction	17	14	14
Transcription	2	2	0
Translation	40	45	14
Transport and catabolism	2	0	1
Xenobiotics biodegradation and metabolism	10	8	5

*VS1, is the comparison of pH 3.0_1 h-VS-pH 4.8_0 h; VS2, is the comparison of pH 4.8_1 h-VS-pH 4.8_0 h; VS3, is the comparison of pH 3.0_1 h-VS-pH 4.8_1 h*.

### Highly induced/suppressed genes

Eighty-eight genes were identified in the comparison VS3, with the limits of (1) *q* < 0.05 and (2) log2FC≥2 (highly induced) or log2FC≤ −2 (highly suppressed) (Supplementary Table 1). Among them, compared to the report of Margalef-Català et al. (2016), some genes showed different changes in their expressions. For example, some genes which were found decrease or increase their expressions in this study were not mentioned in Margalef's report, and others showed opposite expression changes (Supplementary Tables 2, 3). In the Supplementary Table 2, there were some genes shown the same expression patterns between the data from group VS1 and Margalef-Català et al. (2016) but the group VS3 was crosscurrent (orf00275 and orf00218). This again demonstrates the importance of control group (pH 4.8_1 h).

### Main metabolisms modified by acid shock

**Figure 5:**
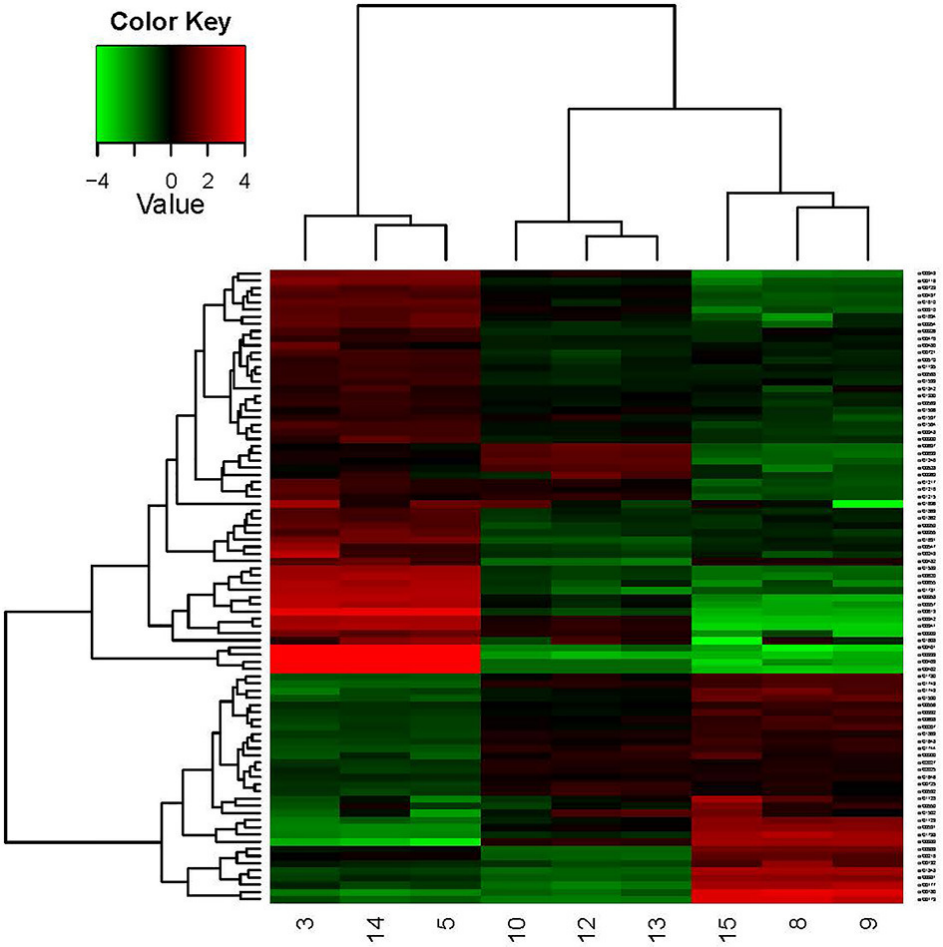
Hierarchical clustering in heat map format of some DEGs shown in Table 4. Each horizontal row represents a differentially expressed gene, whereas each column represents a different growth condition. Green represents downregulated expression and red represents upregulated expression. Log2 values were used to cluster all the DEGs in Java TreeView by hierarchical clustering using Euclidean distance and pairwise average linkage methods. The number 9, 8, and 15 represent the three parallel samples of pH 4.8_0 h; The number 13, 12, and 10 represent the three parallel samples of pH4.8_1h; The number 3, 5,and 14 represent the three parallel samples of pH3.0_1h.

This study was designed to identify genes differentially expressed in pH 3.0 and pH 4.8, by using the RNA-seq technique. The comparisons were carried out between pH 4.8_0 h, pH 4.8_1 h, and pH 3.0_1 h. Table 4 showed the transcriptomic analysis of the relative expression of genes between time 0 and 1 h after the acid shock. The table showed a selection of the most inhibited or promoted genes with known functions. Figure 5 showed the hierarchical clustering in heat map format of DEGs shown in Table 4. From the RNA-seq data, the glycosyltransferase genes related to the carbon source in the medium were over-expressed, while the expression levels of other irrelevant genes were decreased. And the expression of transport proteins, the membrane-like ion transport proteins, amino acid transporter, etc., were significantly increased. These changes are related to the responses of *O. oeni* SD-2a to acid shock.

**Table 4:** Genes in *O. oeni* SD-2a that were significantly expressed under different conditions.

**Related metabolism**	**Gene annotation**	**Gene symbol**	**Relative expression VS1**	**Relative expression VS2**	**Relative expression VS3**
Malate metabolism	3-isopropylmalate dehydrogenase	orf01863	1.6868	−0.0910	1.7500
	2-isopropylmalate synthase	orf01862	1.6778	−0.1969	1.8451
	Malate dehydrogenase	orf00337	6.8475	6.1703	0.6470
	Malate permease	orf00338	6.6973	5.6788	0.9858
	Malate transporter	orf01583	3.9723	0.7612	3.1792
Amino acid transport and metabolism	Chorismate synthase	orf00114	3.5967	0.9001	2.6679
	–	orf00116	3.3990	1.0355	2.3315
	Shikimate kinase	orf00117	3.8605	1.5946	2.2341
	Argininosuccinate synthase	orf00834	2.6672	−0.3068	2.9427
	Argininosuccinate lyase	orf00835	2.2080	0.4201	1.7550
	Proline iminopeptidase	orf01630	1.3972	−2.0444	3.4084
	Aryl-alcohol dehydrogenase	orf00276	−5.0127	−6.3821	1.3383
	4-aminobutyrate aminotransferase	orf00309	3.4839	2.3576	1.0954
	Cystathionine beta-lyase	orf00662	1.0269	−0.6759	1.6741
	Acetolactate synthase	orf01906	1.5036	0.1014	1.3708
Citrate metabolism	CitXG protein	orf00343	3.8526	2.2465	1.5758
	Acetoin reductase	orf00591	−4.1541	−2.0792	−2.1054
	Diacetyl reductase	orf01738	−4.6040	−3.1763	−1.4578
Folate biosynthesis	6-pyruvoyltetrahydropterin synthase	orf01609	1.9045	−0.2666	2.1372
Methane metabolism	Phosphosulfolactate synthase	orf00361	2.8248	−0.2192	3.0117
DNA recombination and repair	Exodeoxyribonuclease VII large subunit	orf01147	−1.1595	0.4337	−1.6236
	DNA recombination protein RecF	orf02025	−1.1990	−0.0298	−1.2001
Cell wall/membrane/envelope biogenesis	D-alanyl-D-alanine carboxypeptidase	orf00619	6.2801	1.6847	4.5623
Nucleotide transport and metabolism	DNA/pantothenate metabolism flavoprotein	orf00828	2.6439	1.2488	1.3628
	Cytidylate kinase	orf00898	−1.1099	−0.1299	−1.0116
	tRNA nucleotidyltransferase	orf00902	−1.2641	−0.1405	−1.1552
	Guanosine monophosphate reductase	orf01030	−1.7991	−3.1500	1.3201
	–	orf01033	−1.1979	−2.2439	1.0180
	DNA polymerase III subunit beta	orf02023	−1.1104	−0.0323	−1.1105
Carbohydrate transport and metabolism	PTS mannose/fructose/sorbose transporter subunit IIC	orf00382	−1.5618	−2.6368	1.0453
	Fructose-bisphosphate aldolase	orf00827	2.8952	1.5427	1.3207
	6-phospho-beta-glucosidase	orf00926	1.0575	−0.4946	1.5227
	2-deoxyribose-5-phosphate aldolase	orf01123	−3.5431	−2.1440	−1.4320
	6-phospho-beta-glucosidase	orf01135	1.1836	−0.0705	1.2204
	PTS sugar transporter subunit IIA	orf01246	1.6351	2.8522	−1.2479
	Transketolase	orf01616	1.0570	−0.2318	1.2573
	PTS ascorbate transporter subunit IIC	orf01617	1.5433	0.3326	1.1833
	–	orf01634	2.7582	1.2510	1.4822
	PTS fructose transporter subunit IIB	orf01636	2.2401	0.8710	1.3435
	Sugar kinase,ribokinase family	orf01748	−3.9058	−0.8487	−3.0952
	Ribose pyranase	orf01749	−2.7958	−1.7178	−1.1114
	–	orf00983	−5.4882	−1.8124	−3.7083
	NADH dehydrogenase	orf00984	−4.4018	−3.2155	−1.2192
	Pyruvate oxidase	orf00829	2.4488	0.7190	1.6978
	Pyruvate,phosphate dikinase	orf01233	1.0998	−0.2212	1.2891
	Aldehyde dehydrogenase	orf00275	−4.7674	−7.6307	2.8333
Stress response	ABC transporter permease	orf00954	2.4217	0.4065	1.9864
	Methionine ABC transporter ATP-binding protein	orf00955	2.0310	−0.2772	2.2728
	ATP-dependent protease	orf01869	−1.4451	−0.2252	−1.2518
	Glutathione S-transferase	orf01108	2.4969	0.5601	1.8986
	Heat-shock protein Hsp20	orf00243	2.6328	−0.0378	2.6468
	F0F1 ATP synthase subunit α	orf00563	1.2636	*No*	*No*
	F0F1 ATP synthase subunit gamma	orf00568	1.3091	−0.0360	1.3118
	F1F0-ATPase subunit beta	orf00569	0.7429	−0.5079	1.2178
	F_*o*_F_1_ ATP synthase subunit epsilon	orf00570	1.1302	−0.2301	1.3277
Translation,ribosomal structure and biogenesis	50S ribosomal protein L19	orf00697	1.4292	2.8198	−1.4228
	DEAD/DEAH box helicase	orf01777	1.4822	2.6878	−1.2358

*VS1, is the comparison of pH 3.0_1 h-VS-pH 4.8_0 h; VS2, is the comparison of pH 4.8_1 h-VS-pH4.8_0 h; VS3, is the comparison of pH 3.0_1 h-VS-pH 4.8_1 h*.

#### Malate and citrate metabolism

One of the strategies that microorganism defense the acid stress is to decrease the internal high concentration proton, as well-known, *O. oeni* can do this by MLF. The way of malate transport into cells is through malate permease (*mleP*), which was up-regulated in this study. Among the MLF, oxidative decarboxylation is an important process. One of the enzymes that catalyzes such a reaction is 3-isopropylmalate dehydrogenase (IPMDH), a member of the β-hydroxyacid oxidative decarboxylase family, to which malate dehydrogenase (decarboxylating) also belong (Pallo et al., 2014). In our study, the 3-isopropylmalate dehydrogenase (*leuB*) and malate dehydrogenase (*maeA*) genes were over-express compares to pH 4.8_0 h, which can offset the influence of low pH in some ways.

Besides, 2-isopropylmalate synthase (*leuA*) and malate transporter gene were also over-expressed. The observed transcriptional activation of *maeA, mleP*, and malate transporter under acid conditions are in accordance with previous studies about wine-related conditions (Augagneur et al., 2007; Costantini et al., 2015; Margalef-Català et al., 2016). But, the expression of *mae*A and *mle*P at pH 3.0_1 h did not have significant differences compared to pH 4.8_1 h, which have not reported before. Citrate lyase is a key enzyme of citrate fermentation, the prosthetic group of citrate lyase is catalyzed by *CitG* and *CitX* in *Escherichia coli*. These two genes are part of the citrate lyase gene cluster, *citCDEFXG* (Schneider et al., 2002). The expression of the citrate lyase operon were induced in this study, which has been previously reported over-expressed under low pH and multi-stress conditions (Olguin et al., 2009; Margalef-Català et al., 2016). The over-expression of genes related to malate transporter and citrate consumption indicated that the consume of L-malate and citrate were associated with acid stress response, and may be as an alternative energy source to sugar metabolism, just as described by Margalef-Català et al. (2016).

Significant changes were also observed within genes involved in diacetyl utilization. Diacetyl is the main aromatic compound associated to MLF and is derived from citrate consumption. Diacetyl reductase and acetoin reductase showed transcriptional inhibition. The expression patterns of these two genes at 1h after acid shock (pH 3.0) were in accordance with previous studies (Margalef-Català et al., 2016). Since there is no subsequent time monitoring, the expression changes are not clear.

#### Amino acid transport and metabolism

As nutrition and flavoring ingredients, amino acids play a key role in the quality of wine. They are the precursors of higher alcohols, esters, and aromatic thiols, which are the flavor active compounds of wine. During wine fermentation, their biosynthetic, and catabolic pathways also play a central role in the biosynthesis and releasing of aroma (Holt et al., 2012).

Chorismate synthase (CS) was over-expressed in VS1 and VS3 comparison group. It catalyzes the biosynthesis of chorismate by 5-enolpyruvylshikimate 3-phosphate. It is the seventh enzyme in the shikimate pathway (SP), and in the biosynthesis of numerous aromatic compounds, the product of this reaction is the last common precursor in bacteria (Macheroux et al., 1999). This reaction catalyzed by CS can release two moles carbonyl, which can combine with free H^+^, decrease the concentration of H^+^. The fifth enzyme of the SP, shikimate kinase (SK), was also over-expressed after acid shock 1 h (Vianna and de Azevedo, 2012). The SP is important for the synthesis of some aromatic amino acids, such as phenylalanine, tyrosine, tryptophan, and other functional aromatic compounds, which will participate in the signaling, electron transport, UV protection, and wound response (Macheroux et al., 1999). These changes can help *O. oeni* to synthesize aromatic compounds and defense the damages caused by acid shock.

Argininosuccinate synthase (ASS) is involved in the biosynthesis of arginine together with argininosuccinate lyase (ASL), and ASS is the rate-limiting enzyme for arginine biosynthesis (Locke et al., 2016). They were over-expressed after acid shock 1 h, which means the up- regulation of arginine synthetase. Arginine can stimulates the expression of some stress-responsive genes, such as ftsH and omrA, and it also can increase the cell number of *O.oeni* at low pH (Arena and de Nadra, 2005; Bourdineaud, 2006). The up-regulation of ASS gene can promote the reproduction of *O. oeni* under stress conditions, for accumulating cell number to resist stress and start MLF.

The enzyme proline iminopeptidase, which releases proline from the N-terminus of small peptides, was over-expressed at 1 h after acid shock. Since peptides account for the largest proportion of total nitrogen in wine (Margalef-Català et al., 2016), it is important for *O.oeni* to utilize them under low pH. Due to the inhibition of proline permease by nitrogen metabolic by-products, during MLF the consume of proline is very few. But the existence of proline can improve the growth of *O.oeni* (Lv, 2012).

The 4-aminobutyrate aminotransferase gene, which transforms gamma aminobutyric acid (GABA) into succinate semialdehyde and L-glutamate, was 3-fold over-expressed after 1 h adaption to acid shock, as reported by Margalef-Català et al. (2016). In bacteria like *Corynebacterium glutamicum* and *E. coli*, GABA can be utilized as the form of carbon and/or nitrogen source, but its assimilation in *O. oeni* is not clear yet (Bartsch et al., 1990; Zhao et al., 2012; Margalef-Català et al., 2016). It is worth a further study.

The cystathionine beta-lyase (CBL) gene was over-expressed after acid shock. CBL is involved in the biosynthesis of methionine. CBL catalyzes the conversion of cystathionine into homocysteine in an α, β-elimination reaction, which will convert to methionine in a later step. The CBL activity plays an important role in aromatic thiol release (Holt et al., 2012). It can improve the flavor and quality of wine during MLF.

Acetolactate synthase (ALS) is the first rate-limiting enzyme for branched-chain amino acid biosynthesis, like valine, leucine, and isoleucine. It converts 2 mol of pyruvate to acetolactate, using thiamine diphosphate (ThDP) as a cofactor (Duggleby and Pang, 2000; Pang et al., 2002; Zheng et al., 2015). Acetolactate can participate in the synthesis pathway of diacetyl (2, 3 - butyl ketone) and its derivatives, which are the main flavor compounds generated by MLF. The up-regulation of ALS gene could help *O. oeni* accomplish the MLF and increase the abundance of aroma compound in wine.

#### Stress response

To alleviate the challenge of reduction in internal pH caused by high concentration proton, the bacteria cytoplasm will be alkalization. Among the efflux systems of harmful-compounds and cell detoxification, one of the important parts is ABC transporters (Leverrier et al., 2004). In this study, there were 121 DEGs detected by RNA-seq related to ABC transporters. Meanwhile, as an important molecular marker of stress response in *O. oeni*, the protein *Hsp*20 was also over-expressed in this essay. ATPase activity has been associated to MLF. Fortier et al. (2003) described the increase of F_0_F_1_-ATPase β subunit mRNA in response to low pH (Fortier et al., 2003). In this work several genes codifying other ATPase subunits (β, γ, and ε) were up-regulated after the acid shock at pH 3.0_1 h (Table 4). However, the F_0_F_1_ ATP synthase subunit α was up-regulated under acid conditions compare to pH 4.8_0 h, but did not have significant differences compare to pH 4.8_1 h. This result is opposite to the report of *O.oeni* PSU-1 under wine like medium (1 h), and similar with the situation after 6 h inoculation (Margalef-Català et al., 2016), meanwhile it indicate that when cells are exposing at low pH, the ATPase activity is increased more quickly than wine like medium, and agrees with the role of this enzyme in the regulation of the cytoplasmic pH and in the acid stress response of *O. oeni*.

The D-alanyl-D-alanine carboxypeptidase (dacC) gene related to cell envelope biogenesis was over-expressed, and it was 6-fold over- expressed at 1 h, and also over-expressed in transcriptomic analysis by Costantini et al. (2015) and Margalef-Català et al. (2016) after adaption with ethanol and WLM. This result is consistent with earlier reports on the barrier and homeostasis functions of cell membranes in the stress response of *O.oeni*, which is well-known (Grandvalet et al., 2008). But previous studies showed that, several genes related to cell wall biosynthesis were significant differentially expressed (Margalef-Català et al., 2016), which point out the relevance of some genes involved in cell wall protection against stress challenges. This point was also confirmed by our study. The expression level of phosphoglycerol transferase gene was significant up-regulated at 1h after acid shock. The dacC gene is involved in the pathway of lipoteichoic acid biosynthesis, and is a part of cell wall biogenesis. The role of cell wall in the stress response of *O.oeni* is worth a further study.

## Conclusions

This is the first transcriptome study using RNA-seq on *O.oeni* under different conditions. The RNA-seq study is useful to identify the metabolisms mostly altered due to low pH conditions. Our results revealed the relevance of carbohydrate metabolism, amino acid metabolism and membrane transport as key metabolisms involved in the adaptation of *O.oeni* SD-2a to acid stress. From GO analysis, the majority of DEGs of all groups (VS1, VS2, and VS3) were found to be involved in the metabolic process, catalytic activity, cellular process and binding. In addition, a considerable proportion of genes are novel, which have a significantly differently expression in this study. These results provide a new viewpoint and crucial resource on the acid stress response in *O. oeni*.

## Author contributions

TW, JS, and HW conceived the idea of the work. LL, HZ and SP designed the experiments and performed the experiments. LL, YL, HL, and HW analyzed the data and wrote the manuscript.

### Conflict of interest statement

The authors declare that the research was conducted in the absence of any commercial or financial relationships that could be construed as a potential conflict of interest.

## Supplementary material

Supplementary Figure 1 Validation of RNA-seq data using RT-qPCR. Eleven representative genes were chosen to validate the RNA-Seq data by RT-qPCR. The black bars represent mean values of log2-transformed fold change obtained from three biological replicates of RT-qPCR with error bars stand for standard deviations. And the red bars represent RNA-Seq data. **(A–E)** Represent gene dnaG, dpoIII, gyrA, gyrB, and ldhD as internal controls, respectively. The number 1–3 represent group pH 3.0_1 h-VS-pH 4.8_0 h, pH 4.8_1 h-VS-pH 4.8_0 h, and pH 3.0_1 h-VS-pH 4.8_1 h, respectively.

Supplementary Figure 2 The scatter diagram of three comparisons.

Supplementary Figure 3 The Volcano Plot of three comparisons.

Supplementary Figure 4 The secondary classification of differentially expressed genes in Gene Ontology (pH 3.0_1 h-VS-pH 4.8_0 h).

Supplementary Figure 5 The secondary classification of differentially expressed genes in Gene Ontology (pH 4.8_1 h-VS-pH 4.8_0 h).

Supplementary Figure 6 The secondary classification of differentially expressed genes in Gene Ontology (pH 3.0_1 h-VS-pH 4.8_1 h).

Supplementary Figure 7 The enrichment of differentially expressed genes by KEGG (pH 3.0_1 h-VS-pH 4.8_0 h).

Supplementary Figure 8 The enrichment of differentially expressed genes by KEGG (pH 3.0_1 h-VS-pH 4.8_1 h).

Supplementary Figure 9 The enrichment of differentially expressed genes by KEGG (pH 4.8_1 h-VS-pH 4.8_0 h).

Supplementary Table 1 Highly induced/suppressed genes in this study.

Supplementary Table 2 Relative expression of genes affected during this study and the research by Margalef-Català et al. (2016) grouped by Clusters of Orthologous Groups (COGs). Samples with different expression pattern are highlighted (yellow).

Supplementary Table 3 Relative expression of genes affected during this study and don't mentioned in the research by Margalef-Català et al. (2016).

## References

[B1] Arena M. E. de Nadra M. C. M. (2005). Influence of ethanol and low pH on arginine and citrulline metabolism in lactic acid bacteria from wine. Res. Microbiol. 156, 858–864. 10.1016/j.resmic.2005.03.010 15939575

[B2] Augagneur Y. Ritt J. F. Linares D. M. Remize F. Tourdot-Marechal R. Garmyn D.. (2007). Dual effect of organic acids as a function of external pH in *Oenococcus oeni*. Arch. Microbiol. 188, 147–157. 10.1007/s00203-007-0230-0 17406856

[B3] Bartsch K. von Johnn-Marteville A. Schulz A. (1990). Molecular analysis of two genes of the *Escherichia coli* gab cluster: nucleotide sequence of the glutamate:succinic semialdehyde transaminase gene (gabT) and characterization of the succinic semialdehyde dehydrogenase gene (gabD). J. Bacteriol. 172, 7035–7042. 10.1128/jb.172.12.7035-7042.1990 2254272 PMC210825

[B4] Beltramo C. Desroche N. Tourdot-Marechal R. Grandvalet C. Guzzo J. (2006). Real-time PCR for characterizing the stress response of *Oenococcus oeni* in a wine-like medium. Res. Microbiol. 157, 267–274. 10.1016/j.resmic.2005.07.006 16171980

[B5] Betteridge A. Grbin P. Jiranek V. (2015). Improving *Oenococcus oeni* to overcome challenges of wine malolactic fermentation. Trends Biotechnol. 33, 547–553. 10.1016/j.tibtech.2015.06.008 26197706

[B6] Bourdineaud J. P. (2006). Both arginine and fructose stimulate pH-independent resistance in the wine bacteria *Oenococcus oeni*. Int. J. Food Microbiol. 107, 274–280. 10.1016/j.ijfoodmicro.2005.09.011 16380184

[B7] Costantini A. Rantsiou K. Majumder A. Jacobsen S. Pessione E. Svensson B.. (2015). Complementing DIGE proteomics and DNA subarray analyses to shed light on *Oenococcus oeni* adaptation to ethanol in wine-simulated conditions. J. Proteomics 123, 114–127. 10.1016/j.jprot.2015.04.019 25920369

[B8] Costantini A. Vaudano E. Rantsiou K. Cocolin L. Garcia-Moruno E. (2011). Quantitative expression analysis of mleP gene and two genes involved in the ABC transport system in *Oenococcus oeni* during rehydration. Appl. Microbiol. Biotechnol. 91, 1601–1609. 10.1007/s00253-011-3498-6 21814807

[B9] Darsonval M. Msadek T. Alexandre H. Grandvalet C. (2016). The Antisense RNA approach: a new application for *in vivo* investigation of the stress response of *Oenococcus oeni*, a Wine-associated lactic acid bacterium. Appl. Environ. Microbiol. 82, 18–26. 10.1128/AEM.02495-15 26452552 PMC4702648

[B10] Desroche N. Beltramo C. Guzzo J. (2005). Determination of an internal control to apply reverse transcription quantitative PCR to study stress response in the lactic acid bacterium *Oenococcus oeni*. J. Microbiol. Methods 60, 325–333. 10.1016/j.mimet.2004.10.010 15649534

[B11] Duggleby R. G. Pang S. S. (2000). Acetohydroxyacid synthase. J. Biochem. Mol. Biol. 33, 1–36.

[B12] Fortier L. C. Tourdot-Marechal R. Divies C. Lee B. H. Guzzo J. (2003). Induction of *Oenococcus oeni* H^+^-ATPase activity and mRNA transcription under acidic conditions. FEMS Microbiol. Lett. 222, 165–169. 10.1016/S0378-1097(03)00299-4 12770702

[B13] Grandvalet C. Assad-Garcia J. S. Chu-Ky S. Tollot M. Guzzo J. Gresti J.. (2008). Changes in membrane lipid composition in ethanol- and acid-adapted *Oenococcus oeni* cells: characterization of the cfa gene by heterologous complementation. Microbiology 154, 2611–2619. 10.1099/mic.0.2007/016238-0 18757795

[B14] Holt S. Cordente A. G. Curtin C. (2012). *Saccharomyces cerevisiae* STR3 and yeast cystathionine beta-lyase enzymes: the potential for engineering increased flavor release. Bioeng. Bugs 3, 178–180. 10.4161/bbug.19566 22572787 PMC3370937

[B15] Kwon S. G. Hwang J. H. Park D. H. Kim T. W. Kang D. G. Kang K. H.. (2016). Identification of differentially expressed genes associated with litter size in Berkshire pig placenta. PLoS ONE 11:e0153311. 10.1371/journal.pone.0153311 27078025 PMC4831801

[B16] Leverrier P. Vissers J. P. Rouault A. Boyaval P. Jan G. (2004). Mass spectrometry proteomic analysis of stress adaptation reveals both common and distinct response pathways in *Propionibacterium freudenreichii*. Arch. Microbiol. 181, 215–230, 497 10.1007/s00203-003-0646-0 14730419

[B17] Li H. Zhao W. Wang H. Li Z. Wang A. (2009). Influence of culture pH on freeze-drying viability of *Oenococcus oeni* and its relationship with fatty acid composition. Food Bioprod. Process. 87, 56–61. 10.1016/j.fbp.2008.06.001

[B18] Li Y. Su J. Yang S. Zhang Y. Li H. (2016). Effect of direct vat set *Oenococcus oeni* SD-2a starter culture on quality of wine. J. Northw. A F Univers.Nat. Sci. Ed. 12, 192–200. 10.13207/j.cnki.jnwafu.2016.12.026

[B19] Liu F. (2002). Study on Enological Characteristics of Selected Oenococcus oeni. Northwest A&F University.

[B20] Liu L. Si L. Meng X. Luo L. (2015). Comparative transcriptomic analysis reveals novel genes and regulatory mechanisms of *Tetragenococcus halophilus* in response to salt stress. J. Ind. Microbiol. Biotechnol. 42, 601–616. 10.1007/s10295-014-1579-0 25563971

[B21] Liu X. Luo Y. Mohamed O. A. Liu D. Wei G. (2014). Global transcriptome analysis of *Mesorhizobium alhagi* CCNWXJ12-2 under salt stress. BMC Microbiol. 14:1. 10.1186/s12866-014-0319-y 25539655 PMC4302635

[B22] Livak K. J. Schmittgen T. D. (2001). Analysis of relative gene expression data using real-time quantitative PCR and the 2(-Delta Delta C(T)) Method. Methods 25, 402–408. 10.1006/meth.2001.1262 11846609

[B23] Locke M. Ghazaly E. Freitas M. O. Mitsinga M. Lattanzio L. Lo Nigro C.. (2016). Inhibition of the polyamine synthesis pathway is synthetically lethal with loss of argininosuccinate synthase 1. Cell Rep. 16, 1604–1613. 10.1016/j.celrep.2016.06.097 27452468 PMC4978703

[B24] Lv Q. (2012). Essential Amino Acid Requirements for Oenococcus oeni Growth and their Effects on Malolatic Malolatic Fermentation. Northwest A&F University.

[B25] Macheroux P. Schmid J. Amrhein N. Schaller A. (1999). A unique reaction in a common pathway: mechanism and function of chorismate synthase in the shikimate pathway. Planta 207, 325–334. 10.1007/s004250050489 9951731

[B26] Margalef-Català M. Isabel A. Albert B. Cristina R. Joaquín B.-G. (2016). Transcriptomic and proteomic analysis of *Oenococcus oeni* adaptation to wine stress conditions. Front. Microbiol. 7:1554. 10.3389/fmicb.2016.01554 27746771 PMC5044463

[B27] Marques A. P. Romão M. V. S. Tenreiro R. (2012). RNA fingerprinting analysis of *Oenococcus oeni* strains under wine conditions. Food Microbiol. 31, 238–245. 10.1016/j.fm.2012.02.006 22608229

[B28] Miller G. Socci N. D. Dhall D. D'Angelica M. DeMatteo R. P. Allen P. J.. (2009). Genome wide analysis and clinical correlation of chromosomal and transcriptional mutations in cancers of the biliary tract. J. Clin. Cancer Res. 28:62. 10.1186/1756-9966-28-62 19435499 PMC2698861

[B29] Olguin N. Bordons A. Reguant C. (2009). Influence of ethanol and pH on the gene expression of the citrate pathway in *Oenococcus oeni*. Food Microbiol. 26, 197–203. 10.1016/j.fm.2008.09.004 19171263

[B30] Olguin N. Champomier-Verges M. Anglade P. Baraige F. Cordero-Otero R. Bordons A.. (2015). Transcriptomic and proteomic analysis of *Oenococcus oeni* PSU-1 response to ethanol shock. Food Microbiol. 51, 87–95. 10.1016/j.fm.2015.05.005 26187832

[B31] Pallo A. Olah J. Graczer E. Merli A. Zavodszky P. Weiss M. S.. (2014). Structural and energetic basis of isopropylmalate dehydrogenase enzyme catalysis. FEBS J. 281, 5063–5076. 10.1111/febs.13044 25211160

[B32] Pang S. S. Duggleby R. G. Guddat L. W. (2002). Crystal structure of yeast acetohydroxyacid synthase: a target for herbicidal inhibitors. J. Mol. Biol. 317, 249–262. 10.1006/jmbi.2001.5419 11902841

[B33] Parreira V. R. Russell K. Athanasiadou S. Prescott J. F. (2016). Comparative transcriptome analysis by RNAseq of necrotic enteritis *Clostridium perfringens* during *in vivo* colonization and *in vitro* conditions. BMC Microbiol 16:186. 10.1186/s12866-016-0792-6 27520106 PMC4983038

[B34] Schneider K. Kastner C. N. Meyer M. Wessel M. Dimroth P. Bott M. (2002). Identification of a gene cluster in Klebsiella pneumoniae which includes *citX*, a gene required for biosynthesis of the citrate lyase prosthetic group. J Bacteriol. 184, 2439–2446. 10.1128/JB.184.9.2439-2446.2002 11948157 PMC134981

[B35] Sorek R. Cossart P. (2010). Prokaryotic transcriptomics: a new view on regulation, physiology and pathogenicity. Nat. Rev. Genet. 11, 9–16. 10.1038/nrg2695 19935729

[B36] Spano G. Massa S. (2006). Environmental stress response in wine lactic acid bacteria: beyond *Bacillus subtilis*. Crit. Rev. Microbiol. 32, 77–86. 10.1080/10408410600709800 16809231

[B37] Sumby K. M. Grbin P. R. Jiranek V. (2012). Validation of the use of multiple internal control genes, and the application of real-time quantitative PCR, to study esterase gene expression in *Oenococcus oeni*. Appl. Microbiol. Biotechnol. 96, 1039–1047. 10.1007/s00253-012-4409-1 23053071

[B38] Trapnell C. Hendrickson D. G. Sauvageau M. Goff L. Rinn J. L. Pachter L. (2013). Differential analysis of gene regulation at transcript resolution with RNA-seq. Nat. Biotechnol. 31:46. 10.1038/nbt.2450 23222703 PMC3869392

[B39] Vianna C. P. de Azevedo W. F. (2012). Identification of new potential *Mycobacterium tuberculosis* shikimate kinase inhibitors through molecular docking simulations. J. Mol. Model. 18, 755–764. 10.1007/s00894-011-1113-5 21594693

[B40] Wang T. Li H. Wang H. Su J. (2015). Multilocus sequence typing and pulsed-field gel electrophoresis analysis of *Oenococcus oeni* from different wine-producing regions of China. Int. J. Food Microbiol. 199, 47–53. 10.1016/j.ijfoodmicro.2015.01.006 25625911

[B41] Wang H. Liu F. Li H. (2003). Effect of malolactic fermentation by different *Oenococcus oeni* strains on amino acid in wine. J. Chin. Inst. Food Sci. Technol. 3, 584, 51–55. 10.16429/j.1009-7848.2003.04.013

[B42] Zhang R. (2008). Study on Performance Trait of Oenococcus oeni SD-2a Active Dry Powder. Northwest A&F University.

[B43] Zhao Z. Ding J. Y. Ma W. H. Zhou N. Y. Liu S. J. (2012). Identification and characterization of gamma-aminobutyric acid uptake system GabPCg (NCgl0464) in *Corynebacterium glutamicum*. Appl. Environ. Microbiol. 78, 2596–2601. 10.1128/AEM.07406-11 22307305 PMC3318837

[B44] Zheng P. Z. Sun X. M. Guo L. L. Shen J. Y. (2015). Cloning, expression, and characterization of an acetolactate synthase (ALS) gene from *Anabaena azotica*. Process Biochem. 50, 1349–1356. 10.1016/j.procbio.2015.05.027

