# Supplementary figures and images for: Transcriptomic Analysis of Oenococcus oeni SD-2a Response to Acid Shock by RNA-Seq
Source: Front Microbiol. 2017 Aug 22;8:1586. doi: 10.3389/fmicb.2017.01586 (PMC5572241; doi:10.3389/fmicb.2017.01586)

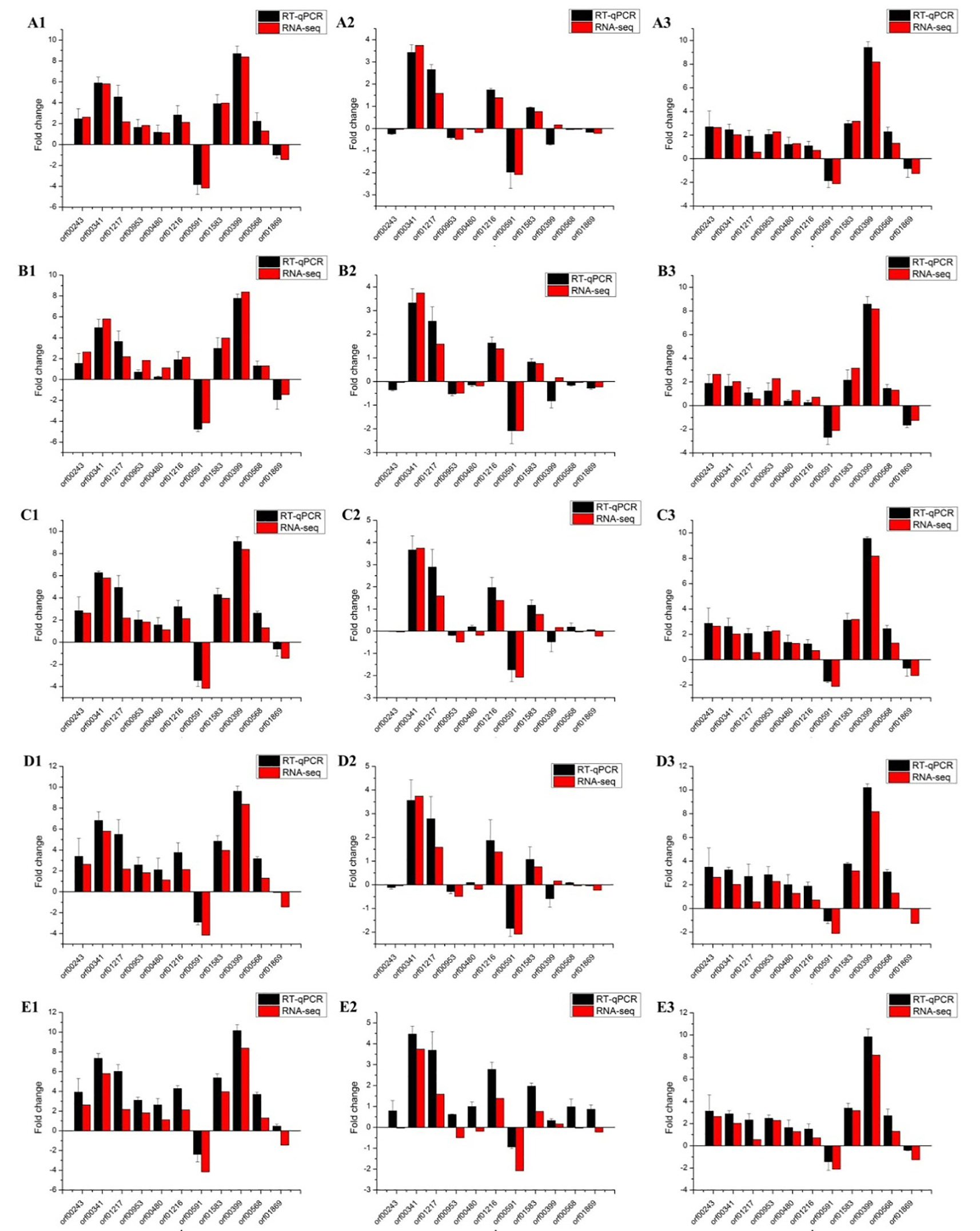

Supplement: Supplementary Figure 1 — Validation of RNA-seq data using RT-qPCR. Eleven representative genes were chosen to validate the RNA-Seq data by RT-qPCR. The black bars represent mean values of log2-transformed fold change obtained from three biological replicates of RT-qPCR with error bars stand for standard deviations. And the red bars represent RNA-Seq data. (A–E) Represent gene dnaG, dpoIII, gyrA, gyrB, and ldhD as internal controls, respectively. The number 1–3 represent group pH 3.0_1 h-VS-pH 4.8_0 h, pH 4.8_1 h-VS-pH 4.8_0 h, and pH 3.0_1 h-VS-pH 4.8_1 h, respectively. [file Image1.JPEG]

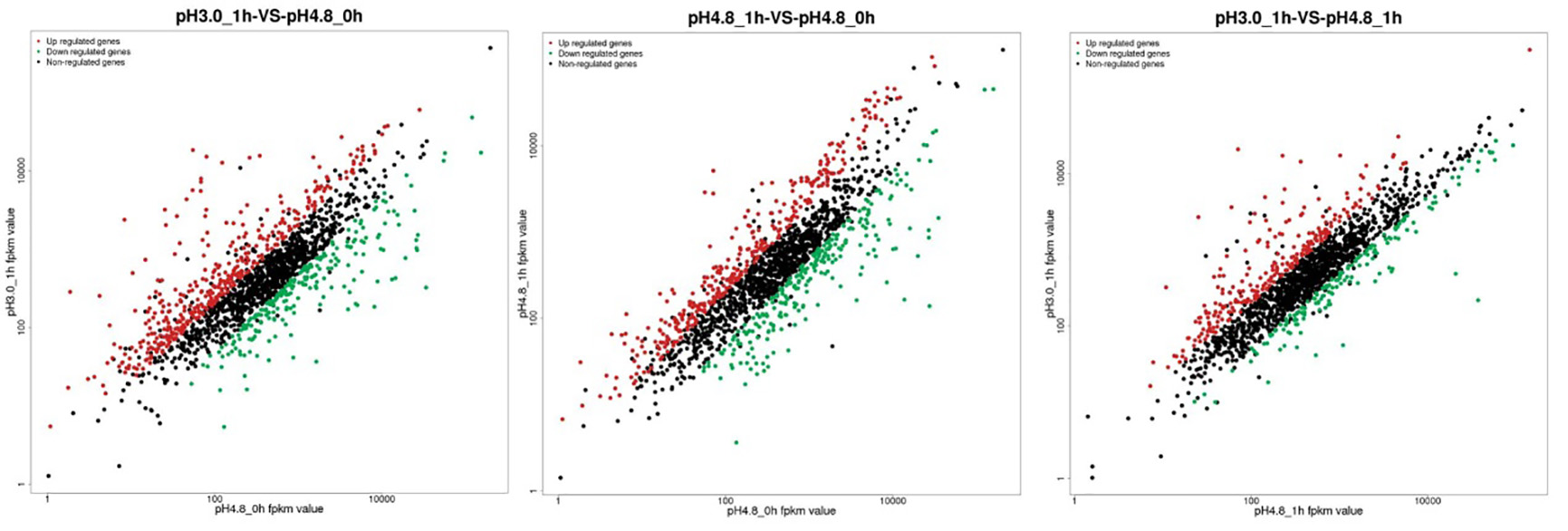

Supplement: Supplementary Figure 2 — The scatter diagram of three comparisons. [file Image2.JPEG]

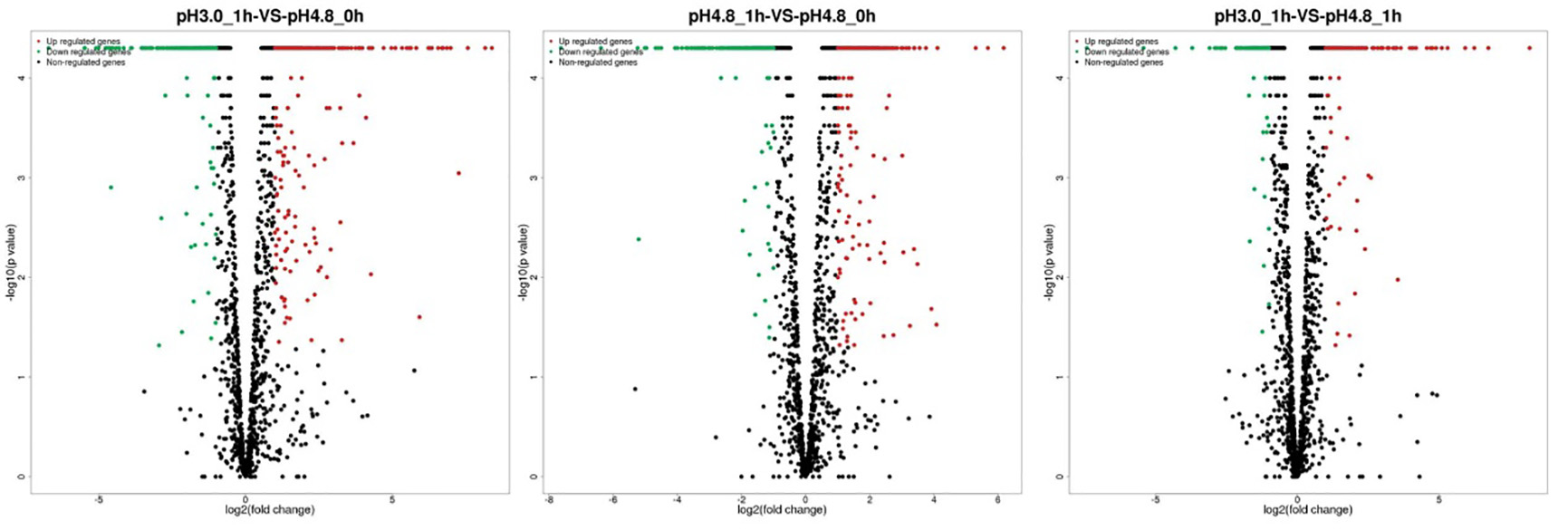

Supplement: Supplementary Figure 3 — The Volcano Plot of three comparisons. [file Image3.JPEG]

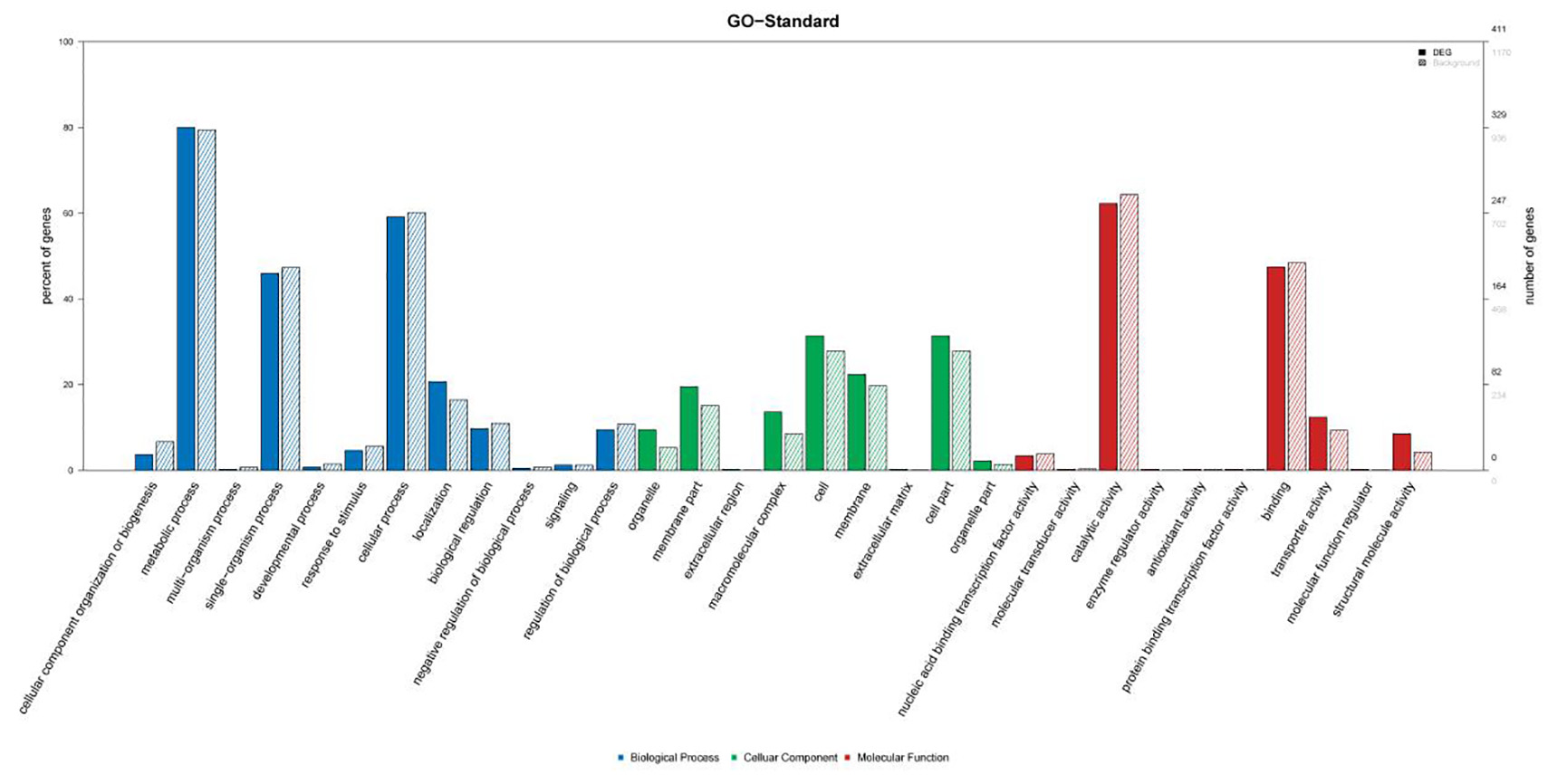

Supplement: Supplementary Figure 4 — The secondary classification of differentially expressed genes in Gene Ontology (pH 3.0_1 h-VS-pH 4.8_0 h). [file Image4.JPEG]

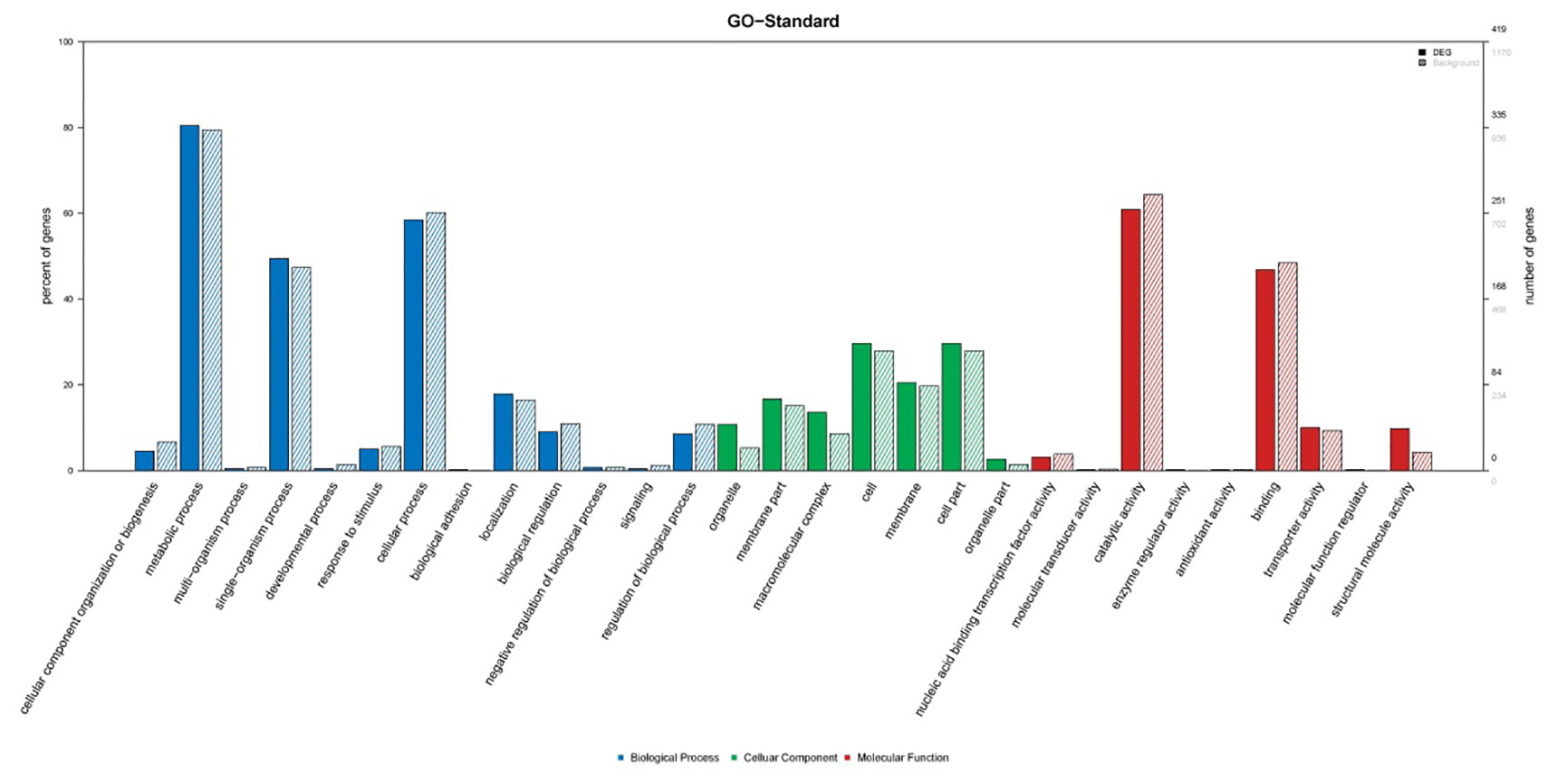

Supplement: Supplementary Figure 5 — The secondary classification of differentially expressed genes in Gene Ontology (pH 4.8_1 h-VS-pH 4.8_0 h). [file Image5.JPEG]

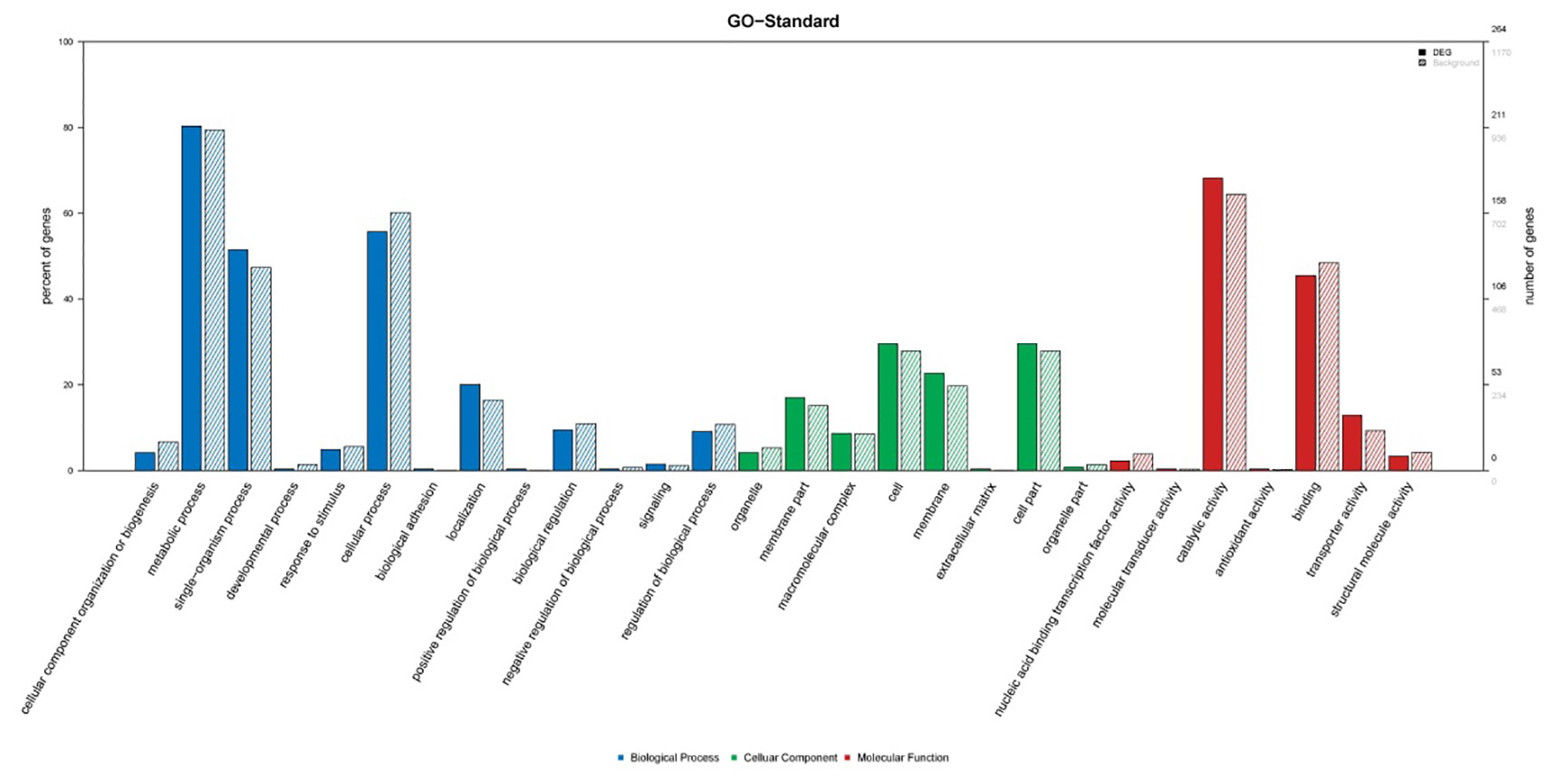

Supplement: Supplementary Figure 6 — The secondary classification of differentially expressed genes in Gene Ontology (pH 3.0_1 h-VS-pH 4.8_1 h). [file Image6.JPEG]

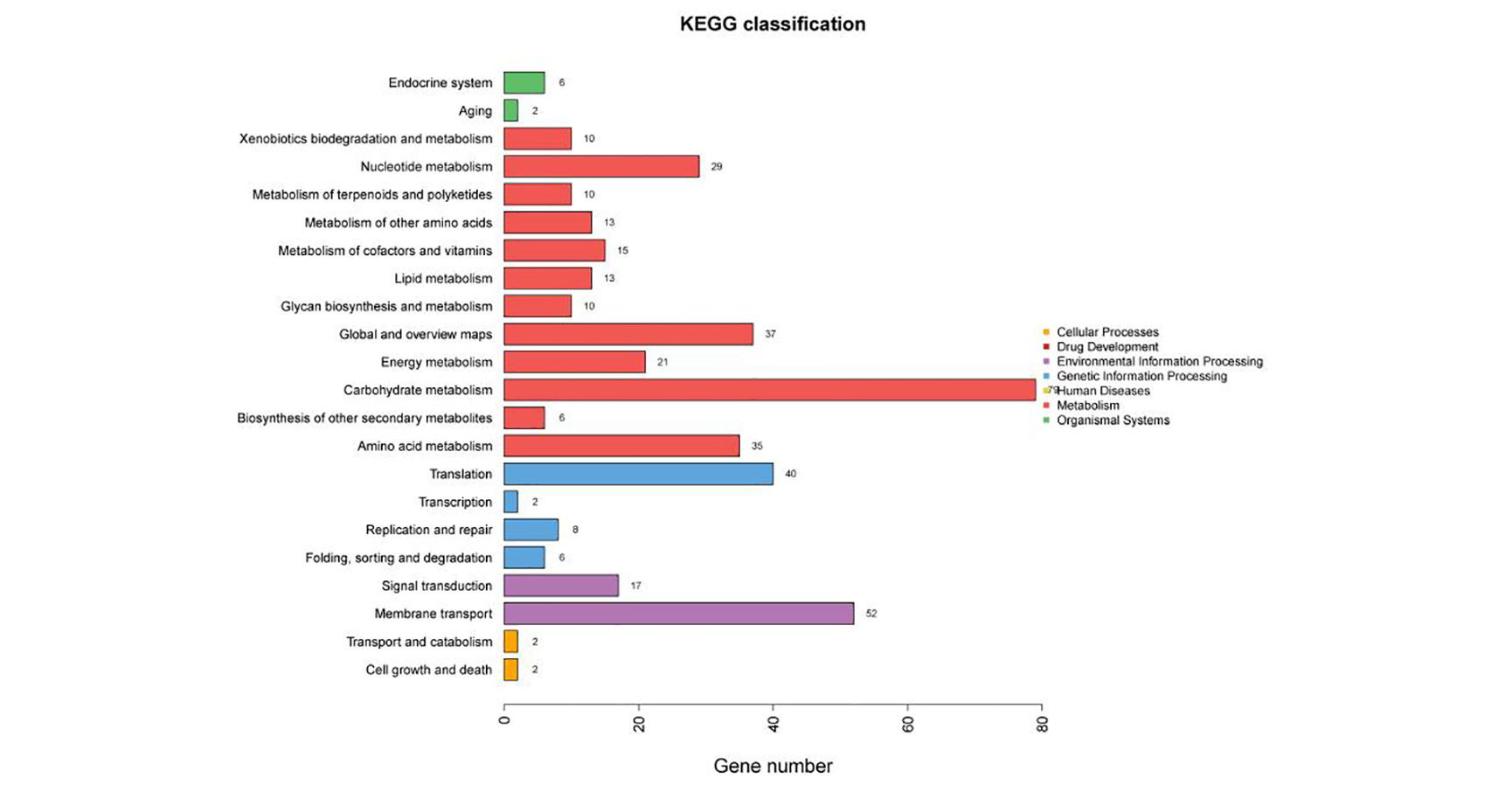

Supplement: Supplementary Figure 7 — The enrichment of differentially expressed genes by KEGG (pH 3.0_1 h-VS-pH 4.8_0 h). [file Image7.JPEG]

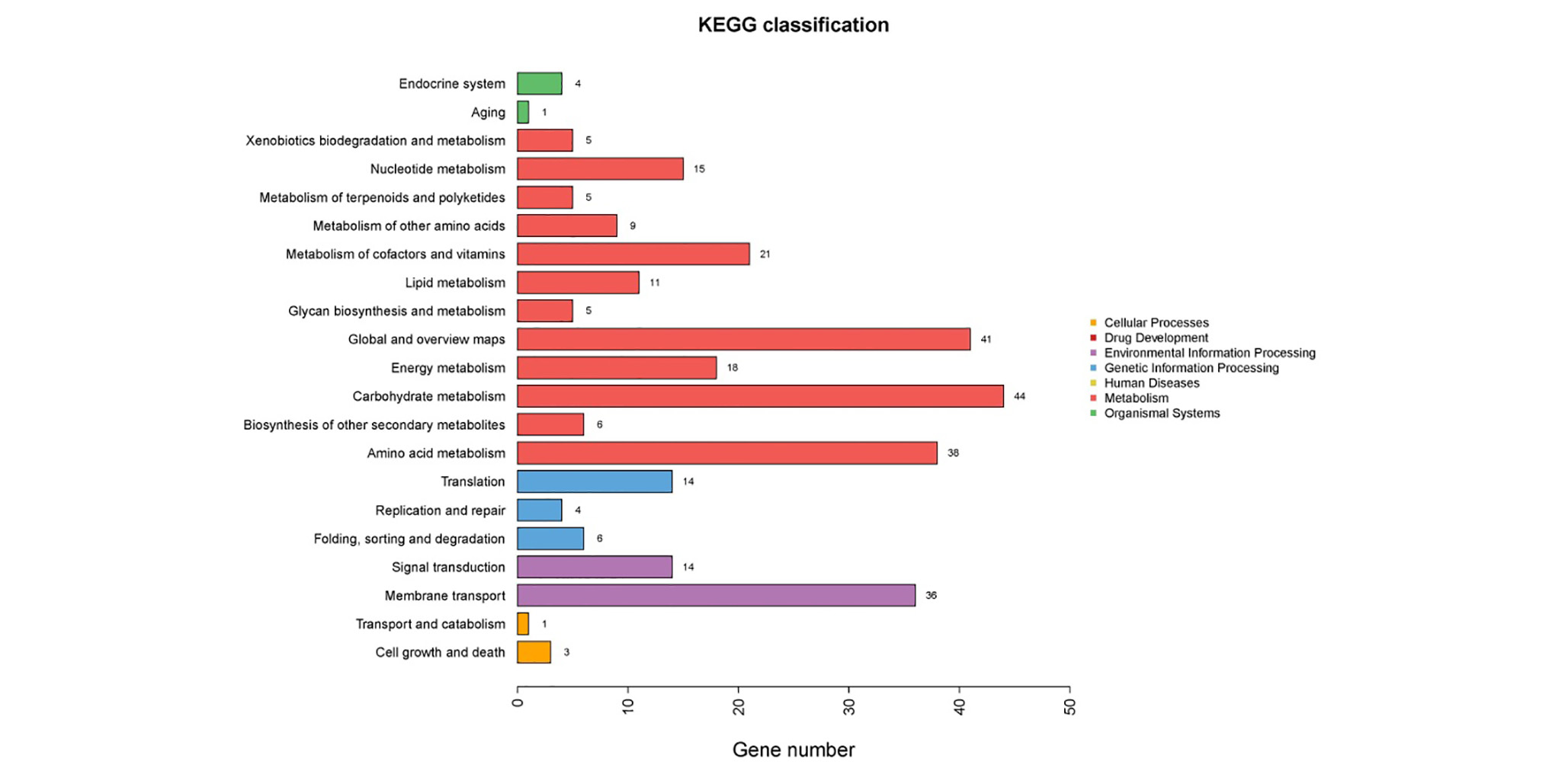

Supplement: Supplementary Figure 8 — The enrichment of differentially expressed genes by KEGG (pH 3.0_1 h-VS-pH 4.8_1 h). [file Image8.JPEG]

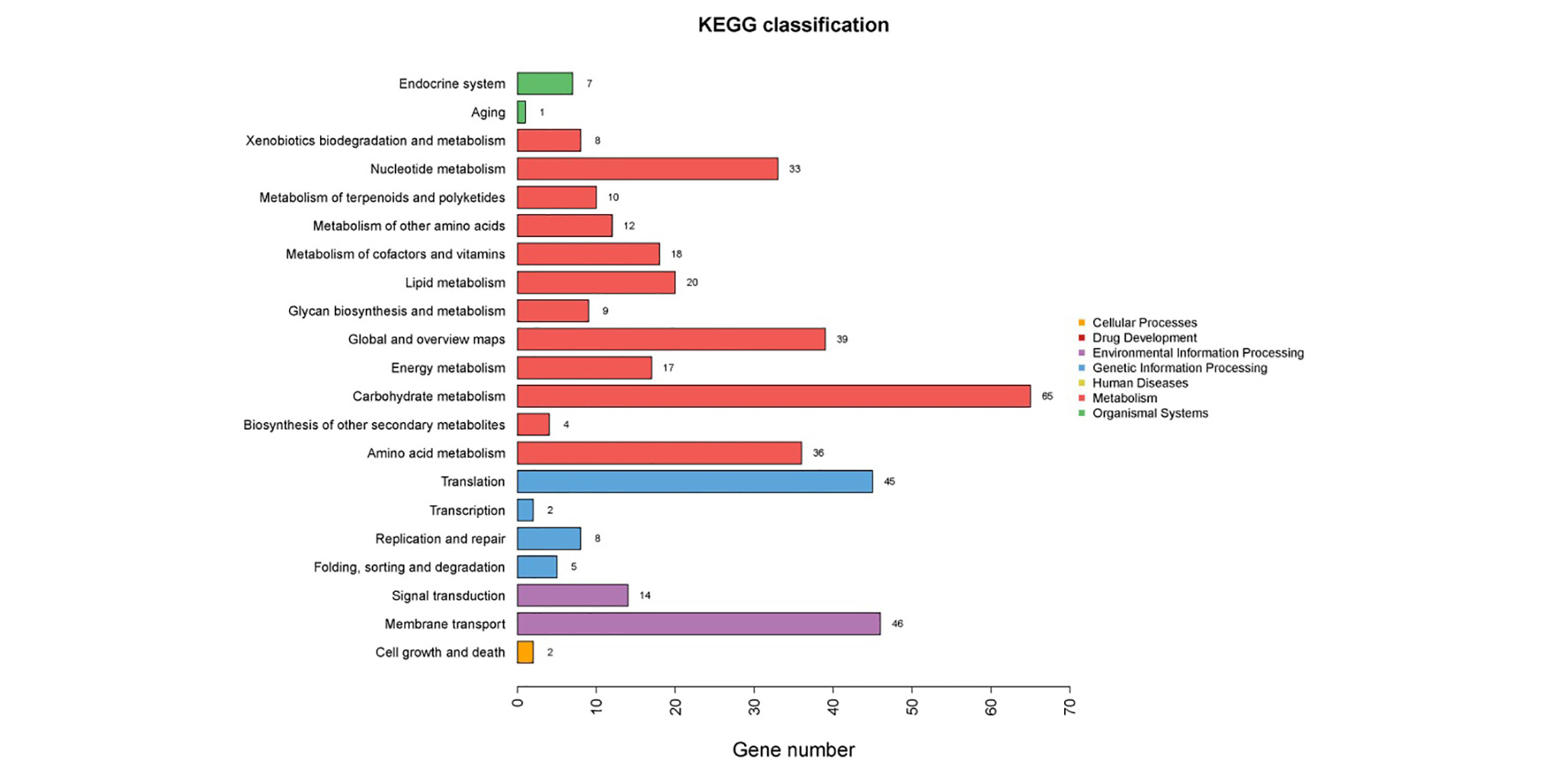

Supplement: Supplementary Figure 9 — The enrichment of differentially expressed genes by KEGG (pH 4.8_1 h-VS-pH 4.8_0 h). [file Image9.JPEG]
