# Supplementary material for: Transcriptomic Analysis of Oenococcus oeni SD-2a Response to Acid Shock by RNA-Seq
Source: Front Microbiol. 2017 Aug 22;8:1586. doi: 10.3389/fmicb.2017.01586 (PMC5572241; doi:10.3389/fmicb.2017.01586)
Supplement: Supplementary Table 1 — Highly induced/suppressed genes in this study. [file Table1.DOCX]

Supplementary Material

Transcriptomic analysis of *Oenococcus oeni* SD-2a response to acid shock by RNA-seq

Longxiang Liu^1^, Hongyu Zhao^1^, Shuai Peng^1^, Tao Wang^4,1^,Jing Su^5,1^,Yanying Liang^1^, Hua Li^1,2,3*^, Hua Wang^1,2,3*^

*** Correspondence:** Hua Li: lihuawine@nwafu.edu.cn
Hua Wang: wanghua@nwsuaf.edu.cn

## Supplementary Tables

**Supplementary Table 1.** Highly induced/suppressed genes in this study

| **Gene symbol** | **Gene annotation** | **Relative expression** | | |
| --- | --- | --- | --- | --- |
|  |  | **VS1** | **VS2** | **VS3** |
| orf01999 | peptidoglycan-binding protein | -4.7248 | 2.6629 | -7.4188 |
| orf01567 | peptidoglycan-binding protein | -5.0912 | 0.3029 | -5.4250 |
| orf00983 | - | -5.4882 | -1.8124 | -3.7083 |
| orf01748 | Sugar kinase,ribokinase family | -3.9058 | -0.8487 | -3.0952 |
| orf01732 | - | -0.4216 | 2.4600 | -2.9107 |
| orf01566 | hydrolase | -1.0778 | 1.7179 | -2.8272 |
| orf01733 | biotin transporter | -0.1910 | 2.5007 | -2.7230 |
| orf01742 | membrane protein | -3.4919 | -0.8671 | -2.6581 |
| orf00600 | transcriptional regulator | -0.3200 | 2.2914 | -2.6543 |
| orf01565 | tRNA-dihydrouridine synthase | -0.8236 | 1.6909 | -2.5458 |
| orf00162 | hypothetical protein | -0.8540 | 1.6010 | -2.5296 |
| orf00716 | hypothetical protein | -1.3999 | 0.9555 | -2.4169 |
| orf00034 | hypothetical protein | -2.0706 | 0.2015 | -2.2770 |
| orf01745 | sugar ABC transporter permease | -2.0037 | 0.1292 | -2.1639 |
| orf00200 | Cyclopentanol dehydrogenase | -6.7084 | -4.5927 | -2.1454 |
| orf01132 | MFS transporter | -2.8867 | -0.8018 | -2.1207 |
| orf01701 | LysR family transcriptional regulator | -2.4343 | -0.3547 | -2.1152 |
| orf00591 | acetoin reductase | -4.1541 | -2.0792 | -2.1054 |
| orf01917 | hypothetical protein | -1.0202 | 1.0116 | -2.0475 |
| orf00993 | membrane-anchored lipoprotein | -4.5352 | -2.5326 | -2.0351 |
| orf01743 | ABC-type uncharacterized transport system,ATPase component | -2.5390 | -0.5678 | -2.0026 |
| orf00399 | membrane protein | 8.3836 | 0.1650 | 8.1852 |
| orf00401 | phospholipid phosphatase | 8.1561 | 1.3945 | 6.7304 |
| orf00402 | glycosyl transferase | 7.5653 | 1.3021 | 6.2339 |
| orf00403 | membrane protein | 6.9787 | 1.0316 | 5.9149 |
| orf00404 | DNA-binding response regulator | 6.6842 | 1.3587 | 5.2933 |
| orf00407 | thiamine biosynthesis protein | 6.5542 | 1.4322 | 5.0898 |
| orf01253 | hypothetical protein,partial | positive_infinity | positive_infinity | 4.9185 |
| orf00400 | cytochrome o ubiquinol oxidase | 7.2562 | 2.3313 | 4.8879 |
| orf00406 | FMN-binding protein | 6.2231 | 1.4323 | 4.7570 |
| orf00589 | hypothetical protein | 3.9758 | -0.8280 | 4.7566 |
| orf00405 | two-component sensor histidine kinase | 6.3371 | 1.6051 | 4.6996 |
| orf00619 | D-alanyl-D-alanine carboxypeptidase | 6.2801 | 1.6847 | 4.5623 |
| orf01260 | hypothetical protein AWRIB429_1356 | positive_infinity | positive_infinity | 4.3049 |
| orf01044 | cell division protein,partial | 2.6326 | -1.6575 | 4.2268 |
| orf00412 | hypothetical protein AWRIB418_1573 | 2.2697 | -2.0056 | 4.2206 |
| orf01781 | ATPase | 3.8995 | -0.3119 | 4.1788 |
| orf01782 | transcriptional regulator | 3.6694 | -0.3963 | 4.0384 |
| orf00408 | Predicted ferric reductase | 5.6282 | 1.6339 | 3.9622 |
| orf01969 | phosphoglycerol transferase | 5.5736 | 1.8957 | 3.6452 |
| orf02053 | hypothetical protein OEOE_0035 | positive_infinity | positive_infinity | 3.6277 |
| orf00622 | - | 4.1472 | 0.5601 | 3.5377 |
| orf01937 | manganese transporter | 4.6985 | 1.1661 | 3.4987 |
| orf00620 | acetylesterase | 4.3119 | 0.7830 | 3.4958 |
| orf00409 | sugar lyase | 4.9628 | 1.4897 | 3.4409 |
| orf01630 | proline iminopeptidase | 1.3972 | -2.0444 | 3.4084 |
| orf00655 | ABC transporter,permease | 4.2661 | 0.8562 | 3.3760 |
| orf01631 | amino acid transporter | 2.3471 | -0.9114 | 3.2274 |
| orf01583 | malate transporter | 3.9723 | 0.7612 | 3.1792 |
| orf00358 | multidrug ABC transporter ATP-binding protein | 5.3269 | 2.1243 | 3.1704 |
| orf00361 | phosphosulfolactate synthase | 2.8248 | -0.2192 | 3.0117 |
| orf00482 | ATP-dependent Clp protease ATP-binding subunit | 0.8514 | -2.1728 | 2.9892 |
| orf01936 | universal stress protein UspA | 4.1931 | 1.1774 | 2.9805 |
| orf00357 | peptide ABC transporter permease | 5.0969 | 2.0982 | 2.9677 |
| orf00198 | hypothetical protein | 0.7437 | -2.2399 | 2.9523 |
| orf00654 | MFS transporter permease | 3.8556 | 0.8829 | 2.9432 |
| orf00834 | argininosuccinate synthase | 2.6672 | -0.3068 | 2.9427 |
| orf01551 | preprotein translocase subunit SecE | positive_infinity | positive_infinity | 2.9116 |
| orf00275 | aldehyde dehydrogenase | -4.7674 | -7.6307 | 2.8333 |
| orf00617 | transcriptional regulator | 3.0191 | 0.2839 | 2.7034 |
| orf00114 | chorismate synthase | 3.5967 | 0.9001 | 2.6679 |
| orf00243 | heat-shock protein Hsp20 | 2.6328 | -0.0378 | 2.6468 |
| orf01981 | hypothetical protein | 2.3939 | -0.2316 | 2.5909 |
| orf01118 | PTS sugar transporter | -1.1826 | -3.7296 | 2.5046 |
| orf00547 | ATP-dependent Clp protease ATP-binding subunit ClpE | 1.9229 | -0.5368 | 2.4330 |
| orf01107 | transcriptional regulator | 2.5575 | 0.1425 | 2.3823 |
| orf01121 | PTS fructose transporter subunit IIB | -1.1691 | -3.5525 | 2.3456 |
| orf00116 | 3-phosphoshikimate 1-carboxyvinyltransferase | 3.3990 | 1.0355 | 2.3315 |
| orf00610 | membrane protein | 1.7985 | -0.5651 | 2.2911 |
| orf00953 | ABC-type metal ion transport system,periplasmic component/surface antigen | 1.8294 | -0.4903 | 2.2877 |
| orf00955 | methionine ABC transporter ATP-binding protein | 2.0310 | -0.2772 | 2.2728 |
| orf01811 | hypothetical protein | 3.6619 | 1.3428 | 2.2668 |
| orf01692 | hypothetical protein | 0.6659 | -1.6216 | 2.2480 |
| orf02044 | sugar ABC transporter permease | 1.1089 | -1.1650 | 2.2388 |
| orf00117 | shikimate kinase | 3.8605 | 1.5946 | 2.2341 |
| orf01984 | hypothetical protein | positive_infinity | positive_infinity | 2.2034 |
| orf02046 | 2-hydroxyacid dehydrogenase | positive_infinity | positive_infinity | 2.1984 |
| orf00241 | D-alanine--poly(phosphoribitol) ligase subunit 2 | 1.8902 | -0.3221 | 2.1810 |
| orf02054 | oxidoreductase ion channel protein IolS | 0.6056 | -1.6028 | 2.1766 |
| orf01797 | N-acetylmuramoyl-L-alanine amidase | 0.3358 | -1.8590 | 2.1676 |
| orf01120 | PTS system,IIC component | -1.0515 | -3.2316 | 2.1473 |
| orf00107 | two-component sensor histidine kinase | 1.2503 | -0.9257 | 2.1447 |
| orf01609 | 6-pyruvoyltetrahydropterin synthase | 1.9045 | -0.2666 | 2.1372 |
| orf00956 | hypothetical protein,GAF domain | 1.3382 | -0.8027 | 2.1064 |
| orf00218 | carbamoyl phosphate synthase small subunit | -1.0981 | -3.2285 | 2.0987 |
| orf00342 | citrate lyase subunit alpha | 5.4859 | 3.3722 | 2.0825 |
| orf00106 | DNA-binding response regulator | 1.2723 | -0.8255 | 2.0662 |
| orf00341 | citrate lyase | 5.8078 | 3.7475 | 2.0288 |
