# Supplementary material for: Transcriptomic Analysis of Oenococcus oeni SD-2a Response to Acid Shock by RNA-Seq
Source: Front Microbiol. 2017 Aug 22;8:1586. doi: 10.3389/fmicb.2017.01586 (PMC5572241; doi:10.3389/fmicb.2017.01586)
Supplement: Supplementary Table 2 — Relative expression of genes affected during this study and the research by Margalef-Català et al. (2016) grouped by Clusters of Orthologous Groups (COGs). Samples with different expression pattern are highlighted (yellow). [file Table2.DOCX]

Supplementary Material

Transcriptomic analysis of *Oenococcus oeni* SD-2a response to acid shock by RNA-seq

Longxiang Liu^1^, Hongyu Zhao^1^, Shuai Peng^1^, Tao Wang^4,1^,Jing Su^5,1^,Yanying Liang^1^, Hua Li^1,2,3*^, Hua Wang^1,2,3*^

*** Correspondence:** Hua Li: lihuawine@nwafu.edu.cn
Hua Wang: wanghua@nwsuaf.edu.cn

## Supplementary Tables

**Supplementary Table 2.** Relative expression of genes affected during this study and the research by Margalef et al.(2016), grouped by Clusters of Orthologous Groups (COGs). Samples with different expression pattern are highlighted (yellow).

| **COGs** |  | **Old locus tag** | **Gene annotation** | **Relative expression in function of time (h)(Margalef-Català et al., 2016)** | | | | | | | **Relative expression from this study** | | |
| --- | --- | --- | --- | --- | --- | --- | --- | --- | --- | --- | --- | --- | --- |
|  |  |  |  | **0.5** | **1** | **2** | **4** | **6** | **8** | **VS1** | | **VS2** | **VS3** |
| C: Energy production and conversion | orf00342 | OEOE_0423 | citrate lyase subunit alpha | 0.73 | 1.13 | 0.93 | 0.59 | 0.54 | 0.64 | 5.4859 | | 3.3722 | 2.0825 |
|  | orf00361 | OEOE_0441 | phosphosulfolactate synthase | 1.01 | 1.62 | 2.22 | 1.99 | 1.73 | 1.66 | 2.8248 | | -0.2192 | 3.0117 |
|  | orf01583 | OEOE_1446 | malate transporter | 3.28 | 4.03 | 4.19 | 3.84 | 3.68 | 3.69 | 3.9723 | | 0.7612 | 3.1792 |
| **E: Amino acid transport and metabolism** | orf00241 | OEOE_0287 | D-alanine--poly(phosphoribitol) ligase | -2.24 | -2.07 | -2.01 | -1.87 | -1.93 | -1.95 | 1.8902 | | -0.3221 | 2.1810 |
|  | orf00357 | OEOE_0438 | peptide ABC transporter permease | 4.02 | 4.40 | 4.48 | 4.19 | 4.00 | 4.07 | 5.0969 | | 2.0982 | 2.9677 |
|  | orf00955 | OEOE_1055 | ABC transporter permease | 1.25 | 0.86 | 0.62 | 0.62 | 0.58 | 0.56 | 2.0310 | | -0.2772 | 2.2728 |
| **G: Carbohydrate transport and metabolism** | orf00275 | OEOE_0324 | aldehyde dehydrogenase | -0.48 | -0.72 | -1.13 | -1.35 | -1.39 | -1.43 | -4.7674 | | -7.6307 | 2.8333 |
|  | orf01745 | OEOE_1609 | sugar ABC transporter permease | -0.38 | -0.52 | -1.03 | -1.23 | -1.22 | -1.21 | -2.0037 | | 0.1292 | -2.1639 |
|  | orf01748 | OEOE_1612 | ribokinase | -1.53 | -1.87 | -2.32 | -2.18 | -1.64 | -1.57 | -3.9058 | | -0.8487 | -3.0952 |
| **H: Coenzyme Metabolism** | orf01609 | OEOE_1473 | 6-pyruvoyl-tetrahydropterin synthase | 1.22 | 1.00 | 0.59 | 0.57 | 0.60 | 0.59 | 1.9045 | | -0.2666 | 2.1372 |
|  | orf01733 | OEOE_1597 | biotin transporter | 0.41 | 0.93 | 0.94 | 1.04 | 0.94 | 0.85 | -0.1910 | | 2.5007 | -2.7230 |
| **I: Lipid transport and metabolism** | orf00401 | OEOE_0485 | phospholipid phosphatase | -1.35 | -1.88 | -1.76 | -1.75 | -1.75 | -1.79 | 8.1561 | | 1.3945 | 6.7304 |
| **K: Transcription** | orf00600 | OEOE_0704 | ArsR family transcriptional regulator | 0.97 | 1.32 | 1.91 | 1.76 | 1.47 | 1.35 | -0.3200 | | 2.2914 | -2.6543 |
|  | orf00617 | OEOE_0718 | transcriptional regulator | 2.09 | 2.68 | 2.49 | 2.01 | 1.78 | 1.61 | 3.0191 | | 0.2839 | 2.7034 |
| **M: Cell wall/membrane/envelope biogenesis** | orf00402 | OEOE_0218 | glycosyltransferase | -1.17 | -1.38 | -1.31 | -1.39 | -1.49 | -1.51 | 7.5653 | | 1.3021 | 6.2339 |
|  | orf00619 | OEOE_0719 | D-alanyl-D-alanine carboxypeptidase | 5.53 | 5.83 | 6.01 | 5.82 | 5.61 | 5.64 | 6.2801 | | 1.6847 | 4.5623 |
|  | orf01567 | OEOE_1430 | peptidoglycan-binding protein | -0.67 | -1.02 | -1.31 | -1.42 | -1.27 | -1.31 | -5.0912 | | 0.3029 | -5.4250 |
|  | orf01999 | OEOE_1839 | peptidoglycan-binding protein | -1.45 | -1.48 | -1.90 | -1.66 | -1.11 | -0.85 | -4.7248 | | 2.6629 | -7.4188 |
| **O: Post-translational modification, protein turnover, and chaperones** | orf00243 | OEOE_0289 | heat-shock protein Hsp20 | -0.92 | -0.43 | -0.55 | -0.89 | -0.88 | -1.13 | 2.6328 | | -0.0378 | 2.6468 |
| **R: General function prediction only** | orf02054 | OEOE_0036 | oxidoreductase ion channel protein IolS | -1.10 | -1.36 | -1.56 | -1.65 | -1.65 | -1.72 | 0.6056 | | -1.6028 | 2.1766 |
|  | orf00200 | OEOE_0242 | 3-β-hydroxysteroid dehydrogenase | -0.84 | -1.07 | -1.38 | -1.48 | -1.50 | -1.52 | -6.7084 | | -4.5927 | -2.1454 |
|  | orf00406 | OEOE_0490 | FMN-binding protein | -0.74 | -0.67 | -0.66 | -0.90 | -0.91 | -1.02 | 6.2231 | | 1.4323 | 4.7570 |
|  | orf00591 | OEOE_0693 | acetoin reductase | -0.96 | -1.32 | -1.53 | -1.80 | -1.82 | -2.01 | -4.1541 | | -2.0792 | -2.1054 |
|  | orf01743 | OEOE_1607 | heme ABC transporter ATP-binding protein | -0.42 | -0.85 | -1.14 | -1.36 | -1.37 | -1.37 | -2.5390 | | -0.5678 | -2.0026 |
| **S: Function unknown** | orf00399 | OEOE_0483 | membrane protein | -2.20 | -2.54 | -2.49 | -2.61 | -2.59 | -2.99 | 8.3836 | | 0.1650 | 8.1852 |
|  | orf00400 | OEOE_0484 | cytochrome O ubiquinol oxidase | -2.05 | -2.42 | -2.40 | -2.59 | -2.40 | -2.69 | 7.2562 | | 2.3313 | 4.8879 |
|  | orf00610 | OEOE_0713 | membrane protein | 2.72 | 3.21 | 3.72 | 3.61 | 3.47 | 3.40 | 1.7985 | | -0.5651 | 2.2911 |
|  | orf01742 | OEOE_1606 | membrane protein | -0.98 | -1.08 | -1.46 | -1.74 | -1.69 | -1.74 | -3.4919 | | -0.8671 | -2.6581 |
| **T: Signal transduction mechanisms** | orf00107 | OEOE_0143 | histidine kinase | 0.82 | 0.93 | 1.02 | 1.13 | 1.01 | 0.96 | 1.2503 | | -0.9257 | 2.1447 |
|  | orf00341 | OEOE_0422 | citrate lyase | 1.25 | 1.50 | 1.25 | 0.95 | 0.91 | 1.07 | 5.8078 | | 3.7475 | 2.0288 |
|  | orf00405 | OEOE_0489 | histidine kinase | -0.75 | -0.84 | -1.22 | -1.32 | -1.24 | -1.23 | 6.3371 | | 1.6051 | 4.6996 |
| **V: Defense mechanisms** | orf00358 | OEOE_0439 | multidrug ABC transporter ATP-binding protein | 3.69 | 4.43 | 4.75 | 4.45 | 4.26 | 4.08 | 5.3269 | | 2.1243 | 3.1704 |
|  | orf00620 | OEOE_0720 | acetyl esterase | 3.23 | 3.72 | 4.21 | 4.03 | 3.69 | 3.61 | 4.3119 | | 0.7830 | 3.4958 |
|  | orf00622 | OEOE_0722 | multidrug ABC transporter ATPase | 1.72 | 1.69 | 2.02 | 2.16 | 1.93 | 2.02 | 4.1472 | | 0.5601 | 3.5377 |
|  | orf00655 | OEOE_0761 | multidrug ABC transporter permease | 0.88 | 1.23 | 1.10 | 1.03 | 1.06 | 1.17 | 4.2661 | | 0.8562 | 3.3760 |
| **Multi COGs** |  |  |  |  |  |  |  |  |  |  | |  |  |
| E F | orf00218 | OEOE_0260 | carbamoyl phosphate synthase small subunit | -1.08 | -1.62 | -1.82 | -2.07 | -2.28 | -2.24 | -1.0981 | | -3.2285 | 2.0987 |
| E V Q | orf00116 | OEOE_0152 | 3-phosphoshikimate 1-carboxyvinyltransferase | 1.71 | 1.92 | 1.62 | 1.49 | 1.64 | 1.95 | 3.3990 | | 1.0355 | 2.3315 |
| E V Q | orf00117 | OEOE_0153 | shikimate kinase | 2.12 | 2.16 | 2.08 | 1.92 | 2.08 | 2.26 | 3.8605 | | 1.5946 | 2.2341 |
| E V Q | orf00834 | OEOE_0939 | argininosuccinate synthase | -0.20 | -0.58 | -0.82 | -0.94 | -1.10 | -1.01 | 2.6672 | | -0.3068 | 2.9427 |
| T K | orf00106 | OEOE_0142 | transcriptional regulator | 1.18 | 1.67 | 1.99 | 2.17 | 1.93 | 1.67 | 1.2723 | | -0.8255 | 2.0662 |
| T K | orf00404 | OEOE_0488 | PhoB family transcriptional regulator | -0.77 | -1.03 | -1.18 | -1.21 | -1.14 | -1.23 | 6.6842 | | 1.3587 | 5.2933 |
