# Supplementary material for: Transcriptomic Analysis of Oenococcus oeni SD-2a Response to Acid Shock by RNA-Seq
Source: Front Microbiol. 2017 Aug 22;8:1586. doi: 10.3389/fmicb.2017.01586 (PMC5572241; doi:10.3389/fmicb.2017.01586)
Supplement: Supplementary Table 3 — Relative expression of genes affected during this study and don't mentioned in the research by Margalef-Català et al. (2016). [file Table3.DOCX]

Supplementary Material

Transcriptomic analysis of *Oenococcus oeni* SD-2a response to acid shock by RNA-seq

Longxiang Liu^1^, Hongyu Zhao^1^, Shuai Peng^1^, Tao Wang^4,1^,Jing Su^5,1^,Yanying Liang^1^, Hua Li^1,2,3*^, Hua Wang^1,2,3*^

*** Correspondence:** Hua Li: lihuawine@nwafu.edu.cn
Hua Wang: wanghua@nwsuaf.edu.cn

## Supplementary Tables

**Supplementary Table 3.** Relative expression of genes affected during this study and don’t mentioned in the research by Margalef et al.(2016)

| **Gene symbol** | **Gene annotation** | **Relative expression** | | |
| --- | --- | --- | --- | --- |
|  |  | **VS1** | **VS2** | **VS3** |
| orf00983 | - | -5.4882 | -1.8124 | -3.7083 |
| orf01732 | - | -0.4216 | 2.4600 | -2.9107 |
| orf01566 | hydrolase | -1.0778 | 1.7179 | -2.8272 |
| orf01565 | tRNA-dihydrouridine synthase | -0.8236 | 1.6909 | -2.5458 |
| orf00716 | hypothetical protein | -1.3999 | 0.9555 | -2.4169 |
| orf00034 | hypothetical protein | -2.0706 | 0.2015 | -2.2770 |
| orf01701 | LysR family transcriptional regulator | -2.4343 | -0.3547 | -2.1152 |
| orf01917 | hypothetical protein | -1.0202 | 1.0116 | -2.0475 |
| orf00993 | membrane-anchored lipoprotein | -4.5352 | -2.5326 | -2.0351 |
| orf00407 | thiamine biosynthesis protein | 6.5542 | 1.4322 | 5.0898 |
| orf01253 | hypothetical protein,partial | positive_infinity | positive_infinity | 4.9185 |
| orf00589 | hypothetical protein | 3.9758 | -0.8280 | 4.7566 |
| orf01044 | cell division protein,partial | 2.6326 | -1.6575 | 4.2268 |
| orf00412 | hypothetical protein AWRIB418_1573 | 2.2697 | -2.0056 | 4.2206 |
| orf01781 | ATPase | 3.8995 | -0.3119 | 4.1788 |
| orf01782 | transcriptional regulator | 3.6694 | -0.3963 | 4.0384 |
| orf00408 | Predicted ferric reductase | 5.6282 | 1.6339 | 3.9622 |
| orf02053 | hypothetical protein OEOE_0035 | positive_infinity | positive_infinity | 3.6277 |
| orf01937 | manganese transporter | 4.6985 | 1.1661 | 3.4987 |
| orf00409 | sugar lyase | 4.9628 | 1.4897 | 3.4409 |
| orf00482 | ATP-dependent Clp protease ATP-binding subunit | 0.8514 | -2.1728 | 2.9892 |
| orf01936 | universal stress protein UspA | 4.1931 | 1.1774 | 2.9805 |
| orf00198 | hypothetical protein | 0.7437 | -2.2399 | 2.9523 |
| orf00654 | MFS transporter permease | 3.8556 | 0.8829 | 2.9432 |
| orf00114 | chorismate synthase | 3.5967 | 0.9001 | 2.6679 |
| orf00547 | ATP-dependent Clp protease ATP-binding subunit ClpE | 1.9229 | -0.5368 | 2.4330 |
| orf00953 | ABC-type metal ion transport system,periplasmic component/surface antigen | 1.8294 | -0.4903 | 2.2877 |
| orf01811 | hypothetical protein | 3.6619 | 1.3428 | 2.2668 |
| orf01692 | hypothetical protein | 0.6659 | -1.6216 | 2.2480 |
| orf02044 | sugar ABC transporter permease | 1.1089 | -1.1650 | 2.2388 |
| orf01797 | N-acetylmuramoyl-L-alanine amidase | 0.3358 | -1.8590 | 2.1676 |
| orf00956 | hypothetical protein,GAF domain | 1.3382 | -0.8027 | 2.1064 |
